# Fluoride and Neurodevelopmental Hazard Modelling: An Assessment of Concentration‐Response Analysis

**DOI:** 10.1111/cdoe.70027

**Published:** 2025-10-31

**Authors:** Jayanth V. Kumar, Mark E. Moss, Honghu Liu, Susan Fisher‐Owens, Andrew Rugg‐Gunn, Julia Kuring

**Affiliations:** ^1^ California Department of Public Health (Retired) Sacramento California USA; ^2^ East Carolina University School of Dental Medicine Greenville North Carolina USA; ^3^ Pediatrics, School of Medicine, Preventive and Restorative Dental Sciences, School of Dentistry University of California San Francisco California USA; ^4^ Public & Population Health University of California Los Angeles California USA; ^5^ Newcastle University Newcastle upon Tyne UK; ^6^ School of Psychology Faculty of Health Sciences The University of Adelaide Adelaide South Australia Australia

**Keywords:** environmental exposures, health policy, maternal and child health, neuroscience/neurology, oral health, public health assessment, public policy, safety, toxicology, water

## Abstract

**Objectives:**

A National Academies Consensus Study report concluded that the evidence did not support an assessment that fluoride is a neurodevelopmental hazard. However, some researchers have undertaken benchmark dose modelling to determine a safe fluoride concentration level in water. Therefore, the suitability of the data for modelling fluoride concentration in urine and water and cognition response using standard criteria was assessed.

**Methods:**

Data quality was evaluated using a standard tool. A random‐effects meta‐analysis of standardised mean difference (SMD) and regression coefficients was conducted to assess effect sizes and heterogeneity. The Environmental Protection Agency (EPA) benchmark dose modelling was utilised to determine the association between fluoride concentrations and cognition scores.

**Results:**

All four maternal urinary fluoride (MUF) studies did not meet the standards for acceptable quality, as identified by the EPA data quality criteria, which are necessary for combining data from different studies for dose–response analysis. The pooled estimate was not statistically significant (*β*_MUF_ = −1.06, 95% CI: −3.63, 1.50; *p* = 0.42; *I* ^2^ = 62%). A meta‐analysis of five studies conducted in fluoridated areas showed a pooled SMD effect size of 0.04 (95% CI: −0.06, 0.14; *p* = 0.42; *I* ^2^ = 0%), favoring higher fluoride. The benchmark dose models did not reveal a functional relationship between MUF or water fluoride concentration and cognitive outcomes (Goodness‐of‐fit *p* < 0.1).

**Conclusions:**

The data quality assessment revealed serious flaws that render the maternal urinary studies unacceptable for hazard assessment and benchmark dose modelling. Therefore, more appropriate studies in endemic fluorosis areas are needed to accurately determine whether fluoride is associated with adverse cognitive outcomes in populations with meaningful exposure.

## 1 Introduction

A U.S. judge in the Northern District of California has instructed the United States Environmental Protection Agency (EPA) to take regulatory action to address the potential IQ deficit risks associated with water fluoridation [1]. The judge expressed that a benchmark analysis result indicating that 0.28 mg/L of fluoride in pregnant women's urine could decrease IQ by 1 point in their children was highly concerning.

To establish a safe level of fluoride (F) in drinking water for the United States, the EPA has set a lower limit of the Benchmark Dose (BMDL) at 1.87 mg/L F, often referred to as the Benchmark Concentration (BMC) and the Benchmark Concentration Lower Limit (BMCL), using severe dental fluorosis as a clinical endpoint [2]. This is a concentration at which no more than 0.5% of exposed children in the susceptible age groups would develop any signs of severe dental fluorosis.

In 2021, a National Academies of Sciences, Engineering and Medicine Consensus Study (NASEM) report reviewed the National Toxicology Program (NTP) draft monograph on a systematic review of the association between fluoride exposure and neurodevelopment and cognition. NASEM concluded that the report fell short of providing a clear and convincing argument that supported the assessment that fluoride is a presumed neurodevelopmental hazard [3]. As a result, the NTP removed the classification of fluoride as a ‘presumed neurodevelopmental hazard’ from its assessment [4].

Several meta‐analyses and reviews have found that the available studies are at moderate to high risk of bias, which limits confidence in a hypothesized causal association [4, 5, 6, 7, 8]. Kumar et al. [6] examined the relationship between fluoride concentration in water or urine and cognition scores in non‐endemic areas (< 1.5 mg/L F) using standardised mean difference meta‐analysis and restricted cubic spline regression analysis. Neither analysis demonstrated a relationship between fluoride concentration and cognition scores in non‐endemic areas [6, 9]. However, another meta‐analysis concluded that there is uncertainty in the dose–response analysis when the fluoride exposure is below 1.5 mg/L F [10].

Grandjean et al. [11] conducted benchmark dose modelling to identify a safe fluoride level based on three secondary data cohort studies. The combined analysis revealed a BMC of 0.47 mg/L urinary F (BMCL, 0.28 mg/L) for a 1‐point change in IQ, indicating an adverse effect. However, instead of using the reported positive coefficient for the Odense Child Cohort (OCC) study (*β* = 0.08; 95% CI: −1.14, 1.30 or *β* = 0.18; 95% CI: −0.39, 1.76 for a doubling in exposure), the authors selected a negative coefficient (*β* = −0.94; *p* = 0.43) to determine if the merged datasets represent a homogeneous picture of a dose–response for their BMC analysis. According to the authors, if the estimated concentration–response is increasing, the BMC is not defined [12]. The authors used summary regression coefficient data associated with maternal urinary fluoride (MUF) and the child's intelligence or cognition score from three cohort studies (OCC, Early Life Exposures in Mexico to Environmental Toxicants (ELEMENT) project, Mexico, and Maternal–Infant Research on Environmental Chemicals (MIREC) program, Canada) and omitted the INMA (INfancia y Medio Ambiente—Environment and Childhood) birth cohort study from Spain [11, 13, 14, 15].

The EPA has developed guidance, including a Benchmark Dose Response software for risk assessment [16]. The criteria for combining data from multiple studies for a BMD calculation state, ‘Datasets that are statistically and biologically compatible may be combined prior to dose‐response modeling… If it is plausible that the multiple datasets represent a homogeneous picture of the dose‐response (for example, the responses at doses common to two or more datasets are essentially the same and statistically undifferentiable), then this is a justifiable approach’.

Furthermore, the EPA has outlined principles to guide the series of steps and processes for incorporating systematic review approaches and methods into Toxic Substances Control Act (TSCA) risk evaluations, specifically evaluating data quality and its appropriateness [17]. Therefore, the aim of the study was to assess the suitability of available cohort studies for modelling fluoride concentration in urine and water in relation to IQ response by examining the similarities between samples, average responses at similar concentrations, the homogeneity of effect sizes, and benchmark concentration model fit.

## 2 Methods

Six cohort studies were identified from the literature searches conducted by NTP. Data extraction results obtained by NTP are publicly available and downloadable (https://hawcproject.org/assessment/405/). In addition, a 2024 study from.

Australia [18] was identified through PubMed, Mendeley, and Google Scholar. Two authors abstracted data from the eligible studies using a standard form. The details of the data extraction procedures have been reported before [6]. For this analysis, all four studies that used MUF at the individual level as an exposure variable were selected as well as three additional cohort studies that used water fluoride concentration measured at the community level (Figure SA) [11, 13, 14, 15, 18, 19, 20, 21].

### 2.1 Data Quality Assessment

Two authors assessed the study quality using the method developed by the EPA [17] to determine if serious flaws would make epidemiological studies unacceptable for hazard assessment (Table SA).

### 2.2 Data Analysis

As raw data from these studies were unavailable, the summary data (mean IQ or cognition scores and regression coefficients associated with urinary F concentration) were used along with other relevant sample characteristics to determine if the results from four urinary F cohort studies and water fluoridation studies present a homogenous picture. A standard random‐effects model meta‐analysis approach was used to assess the heterogeneity of effect sizes derived from standardised mean scores and regression coefficients. The details of this meta‐analysis method have been discussed before [6].

The US EPA BMDS Desktop was used to conduct a dose–response analysis of continuous data using summary data (number of subjects, MUF concentration, mean IQ or cognition score, and standard deviation) [22]. One study did not provide mean IQ scores for lower and higher fluoride groups [11]. Therefore, 803 data points (out of 837) were extracted from the published graph, using WebPlotDigitizer, a data extraction tool [23]. The mean IQ and MUF values derived from data extraction were similar to those in the published paper (mean IQ 98.9 [SD 12.8] vs. 99.44 [SD 12.34]) for the derived data and the published study, respectively, and the mean MUF was 0.59 (SD 0.32; range 0.08–3.05, with a median of 0.52). The median MUF was the cutoff to create lower and higher fluoride groups (Figure SB). In addition, the linear regression coefficient (*β* = 0.26, 95% CI: −2.02, 2.54) reported by Taylor et al. (NTP) was used for the OCC study [10].

The benchmark dose model assessed the statistical association between maternal urinary and water fluoride concentrations and mean cognition scores. The scatter diagrams were visually inspected. The linear model was compared directly with that selected by Grandjean et al. [11] Although the EPA recommends a one‐standard‐deviation (SD) change in the benchmark response for the BMC analysis, a 0.5 SD change was used to be conservative in this assessment to ensure a higher level of safety.

## 3 Results

### 3.1 Characteristics of the Sample

The sample characteristics varied substantially among the four maternal urinary fluoride studies (Table 1). The percentage with greater than high school education varied from a low of 10% in the ELEMENT cohorts to more than 76% in the MIREC and OCC cohorts. Similarly, smoking rates varied markedly from 1% to 48%. The lowest MUF concentration groups exhibited a 10‐point variation in their mean response scores, ranging from 98 at 0.37 MUF in the OCC cohort to 108 at 0.4 MUF in the MIREC cohort. Although the MIREC and OCC cohorts had similar levels of education (> 68% with high school education), the mean response scores varied by 8 points on the Wechsler Intelligence Scales at approximately 0.5 mg/L MUF.

**TABLE 1:** Selected characteristics of the sample from seven cohort studies.

Study and country	Setting	F Level	F Conc (mg/L), (MUF) and adjustment procedure	N	IQ/Cognition Index	Age	Mean and regression coefficient (95% CI)	SD	Smokers (%)	>High School Education (%)	HOME Score or Income
OCC, Denmark (Grandjean et al.)^a^	Naturally occurring F	All	0.58 adjusted for the creatinine concentration	837	Danish version of the abbreviated Wechsler Intelligence Scales for Children	7	99.4 *β* = 0.26, (−2.02 to 2.54) As reported by Taylor et al.	12.34	14.4	76.8	NA
OCC (estimated)		Lower Higher	0.37 0.81	408 395			98.1 99.8	13.3 12.2			
MIREC, Canada (Green et al.)		Combined	0.51 adjusted MUF for specific gravity (SG)	512	Wechsler Primary and Preschool Scale of Intelligence‐III	3–4	107.16	13.26	2	68	47.32
MIREC NF	Non‐fluoridated Water F = 0.13	Lower	0.40	238			108.07	13.31	3	66	47.28
MIREC F	Fluoridated Water F = 0.59	Higher	0.69	162			108.21 *β* = −2.01 *p* = 0.16 (Grandjean et al.)	13.72	1	76	48.14
ELEMENT, Mexico ELEMENT IQ (Low F) Bashash et al. (Used in the BMC analysis)	Salt	Lower	0.54 adjusted for creatinine	77	Wechsler Abbreviated Scale of Intelligence (WASI)	6–12	95.37	10.31	48	Years of Educ 10.8 ± 2.85	35.54
ELEMENT IQ (High F)	Salt	Higher	1.01	112			96.8	11.16			35.54
ELEMENT (GCI and IQ)											
Goodman et al. (Used in the MUF meta‐analysis)	Salt	Combined	MUF	Age 4 = 386 5 = 308 6–12 = 278	4, 5 – McCarthy Scales of Children's Abilities (MSCA) 6–12 – WASI	4 5 6–12	96.58 96.62 96.20 *β* = −4.09 0.009 (Grandjean et al.)	13.96 12.52 11.12	48.96 51.30 49.64	Years of Educ 10.77 ± 2.83 10.61 ± 2.87 10.91 ± 2.87	
INMA NF, Spain Ibarluzzea et al.	Non‐fluoridated water	Lower	0.45 MUF levels adjusted for creatinine	123	McCarthy Scales of Children's Abilities (MSCA)	4.4 ± 0.1	98.67	15.7	19.8	48	NA
INMA F Water F 0.81 vs. < 0.1	Fluoridated water	Higher	0.82	124			101.47 *β* = 3.37 (−2.09 to 8.83)	15.5	17.21	53	NA
Water F exposure											
Dunedin Multidisciplinary Health and Development Study, New Zealand Broadbent et al.	Non‐ fluoridated water	Lower	0.0–0.3 mg/L Midpoint 0.15	99	Weschler Adult Intelligence Scale‐Revised (WISC‐R)	Assessments at age 5,7,9,11,13	99.8	14.5	—	—	—
	Fluoridated water	Higher	0.7–1.0 mg/L Midpoint 0.85	891			100.0 b_CWF_ = –0.14 (–3.49 to 3.20); Ref no F.	15.1			
APrON study, Canada Dewey et al.	Non‐ fluoridated water	Lower	0.1 to 0.4 mg/L Midpoint 0.25	101	Weschler Preschool and Primary Scale of Intelligence (WPPSI‐IV)	3–5 years	104.62	11.41	13.86	81.19	Household income > 70k CAD 83.17%
	Fluoridated water	Higher	0.7 mg/L	295			104.69 b_CWF_ = 0.36 (−2.69 to 3.41); Ref no F	14.02	27.12	75.16	83.12%
National Child Oral Health Study, Australia Do et al.	Non‐ fluoridated water	Lower	< 0.3 mg/L Midpoint 0.16	68	WAIS ‐IV	16–26 years (Mean 19.6, SD = 2.3)	108.6		NA	77.6	Low income 15.3%
	Fluoridated water	Higher	> 0.7–1.01 mg/L Midpoint 0.86	194			109.1 *β*_CWF_ = 1.12 (−2.81 to 5.05); Ref no F.			86.1	Low income 20.6%

^a^ For the OCC study, the mean IQ and STD were estimated using the extracted data from a graph. The percent > High school education level was abstracted from Beck et al. https://doi.org/10.1093/aje/kwad110.

### 3.2 Data Quality Assessment

None of the four MUF studies met the standards for acceptable quality identified by the EPA data quality criteria (Table S1). The reported information indicates that the selection of study participants and the analysis sample in the ELEMENT and MIREC studies do not represent the population, as they are based on a non‐probability cluster sample. The measurement of fetal fluoride exposure using maternal spot urinary fluoride as a biomarker was based on less than three samples during pregnancy, and the adjustment for urinary fluoride dilution varied among the studies. On the other hand, the measurement of fluoride in drinking water for the five cohorts was based on multiple fluoride measurements. It was classified as having high confidence in data quality. The outcome measurement varied substantially among the studies, and inter‐examiner reliability data were not provided except for one ELEMENT subcohort out of their four cohorts. The data analytical strategy in the MIREC, ELEMENT, and OCC studies aligned more closely with a prediction model than a causal inference model, where covariate selection was driven more by estimating an exposure‐outcome relationship rather than overall consideration of parameters related to model fit.

### 3.3 Meta‐Analysis

#### 3.3.1 Maternal Urinary F and Regression Coefficient Analysis

Table 2 presents the meta‐analysis of maternal urinary fluoride‐associated regression coefficients. It shows that the pooled effect size *β* decreased from −2.07 (95% CI: −3.61, −0.52; *p* = 0.009; *I* ^2^ = 22%) as reported by Grandjean et al. [11] to a weaker and statistically not significant association (−1.78, 95% CI: −4.28, 0.73; *p* = 0.16; *I* ^2^ = 61%) when the negative coefficient (−0.94) was replaced with the positive OCC coefficient (0.26). There was substantial heterogeneity, indicating no common effect across studies and therefore undermining the validity of the synthesised estimate. In the leave‐one‐out analysis, a pooled effect size β ranged from −0.13 (95% CI: −2.51, 2.25; *p* = 0.92) to −1.78 (95% CI: −4.28, 0.73; *p* = 0.16), and the greatest difference in results was observed when excluding the salt fluoridation exposure study. This association effect of a −0.065 IQ point decrease (not statistically significant) for the 0.5 mg/L increase in MUF from non‐fluoridated to fluoridated communities is negligible. Figure SC shows the details.

**TABLE 2:** Meta‐analysis showing pooled estimates and heterogeneity for maternal urinary fluoride effects by replacing the negative coefficient with the positive OCC regression coefficient from the full cohort. Fixed or random effects analysis of regression coefficients associated with 1 mg/L increase in maternal urinary fluoride.

Studies	OCC coefficient (95% confidence interval)^a^	Model	Pooled β overall effect and 95% confidence interval^b^	Heterogeneity: *I* ^2^
Grandjean et al. [12] (OCC negative coefficient from the Table S2)	−0.94 (−3.27, 1.39)	Fixed effects	−2.07 [−3.61, −0.52] Test for overall effect: *p* = 0.009	*I* ^2^ = 22%; *p* = 0.28
Revised by replacing with the positive OCC coefficient (from Taylor et al.) [11]	0.26 (−2.02, 2.54)	Random effects	−1.78 [−4.28, 0.73] Test for overall effect: *p* = 0.16	*I* ^2^ = 61%; *p* = 0.08
Including the INMA cohort study			−1.06 [−3.63, 1.50] Test for overall effect: *p* = 0.42	*I* ^2^ = 62%; *p* = 0.05
Excluding the salt fluoridation exposure ELEMENT study by Goodman et al.			−0.13 [−2.51, 2.25] Test for overall effect: *p* = 0.92	*I* ^2^ = 41%; *p* = 0.18

^a^ The *Cochrane Handbook for Systematic Reviews of Interventions* guidance was used to convert *p* values to standard errors.

^b^ The calculation is based on 1 mg/L F increase. However, the difference in MUF between fluoridated and non‐fluoridated cities in the Canadian study was about 0.5 mg/L.

#### 3.3.2 Water Fluoridation and Standardised Mean Difference Analysis

**FIGURE 1:**
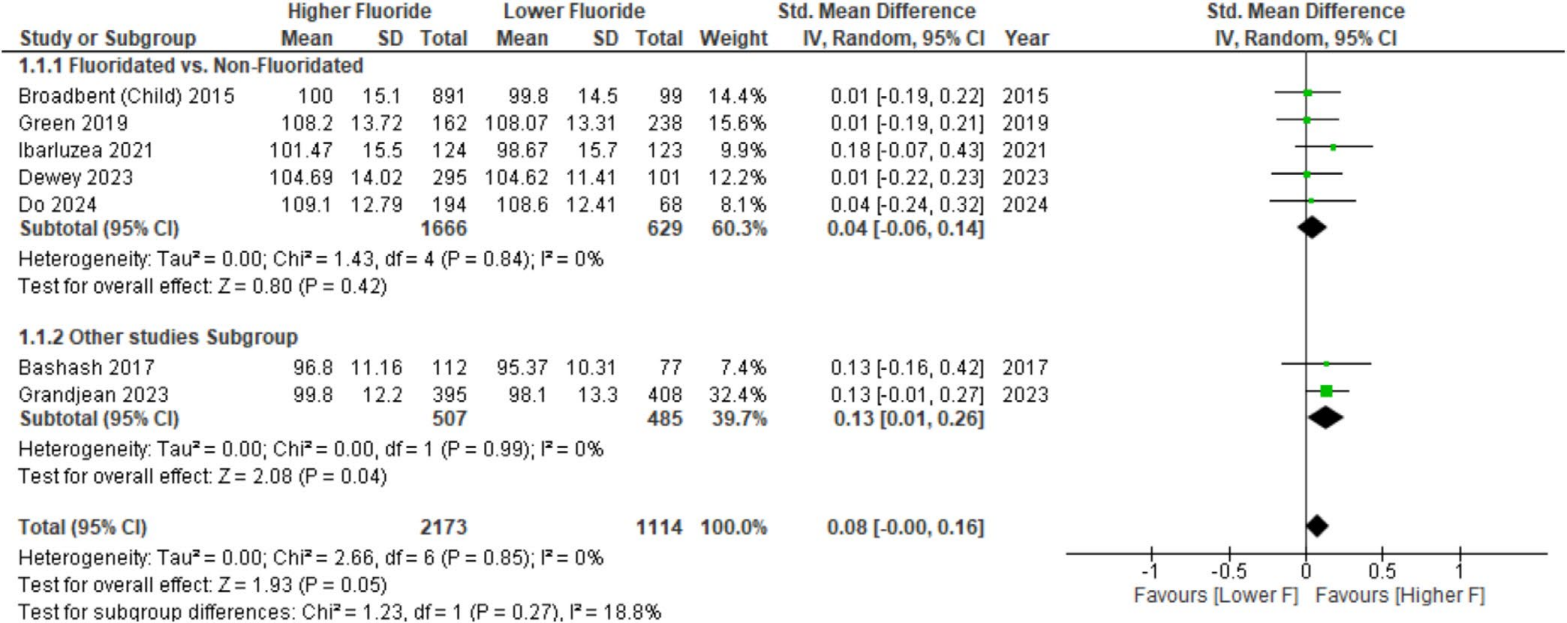
Random effects analysis of standardised mean difference (SMD) and 95% CI of children's IQ score associated with exposure to higher fluoride. For each study, squares represent the point estimate, and the horizontal line shows the 95% CIs. Solid diamonds show the pooled estimate. The *I* ^2^ and *p* values for heterogeneity and test for overall effect are shown.

A standardised mean difference meta‐analysis of five studies comparing fluoridated and non‐fluoridated areas showed that the pooled SMD effect size of 0.04 (95% CI: −0.06, 0.14; *p* = 0.42), favouring higher F, was not statistically significant (Figure 1). Furthermore, no heterogeneity was observed (I^2^ = 0%; *p* = 0.84). When all seven studies were considered, the pooled estimate became marginally significant, favouring higher F exposure (SMD = 0.08, 95% CI: 0.00, 0.16; *p* = 0.05).

### 3.4 Benchmark Concentration Analysis

#### 3.4.1 Maternal Urinary Fluoride and IQ/GCI Scores

**FIGURE 2:**
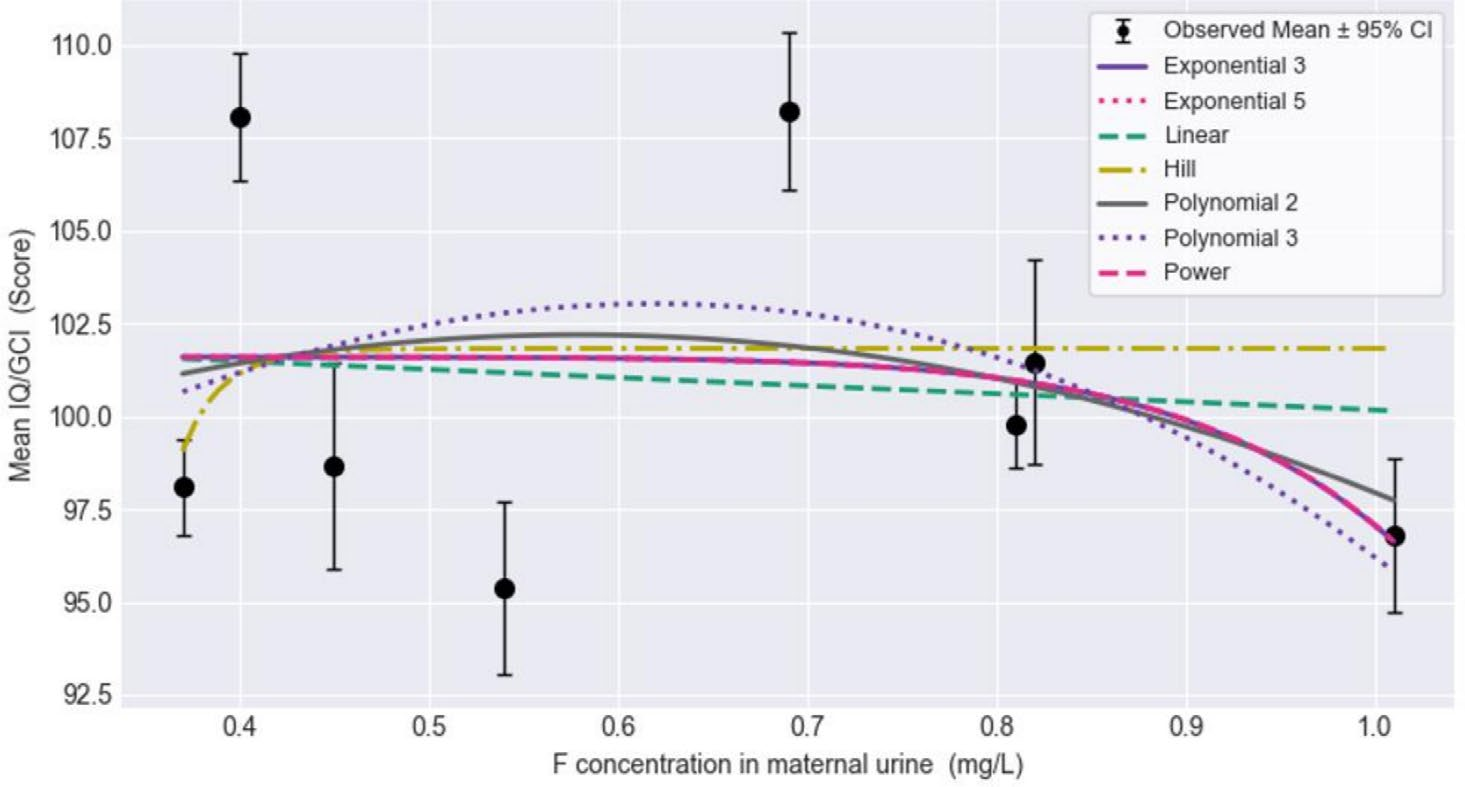
Benchmark dose models for spot maternal urinary fluoride concentration and cognitive function outcomes.

All concentration‐response models were either unusable or questionable (Figure 2). A visual inspection of the mean IQ/GCI summary data (Figure SD) did not reveal a functional relationship between MUF and intelligence outcome in this exposure range (mean 0.37–1.01). At the lowest concentration of about 0.4 mg/L MUF, the difference in cognition scores between the OCC and MIREC studies was 10 points. The difference in mean IQ scores between the lowest exposed group from OCC (0.37 mg/L F) and the highest exposed group in ELEMENT (1.01 mg/L F) was not statistically significant (mean IQ difference −1.3; 95% CI: −3.9, 1.3; *p* = 0.34). A linear model (BMR = 0.5 STD) based on three studies showed a BMD of 19.9 mg/L F and a BMDL of 2.0. The Global Goodness‐of‐fit Test, which measures how the model‐predicted dose‐group response differs from the observed response, indicated a poor fit (*p*‐value < 0.1). The scaled residuals > 2 suggested poor local fit. The addition of the ELEMENT cohort showed a lower BMD (3.1 mg/L) and BMDL (1.4 mg/L); however, it did not alter the interpretation of the results. All models were unusable when 1 IQ point was used for BMR in a sensitivity analysis (Figure SD).

#### 3.4.2 Water Fluoride Concentration and IQ/GCI Scores

**FIGURE 3:**
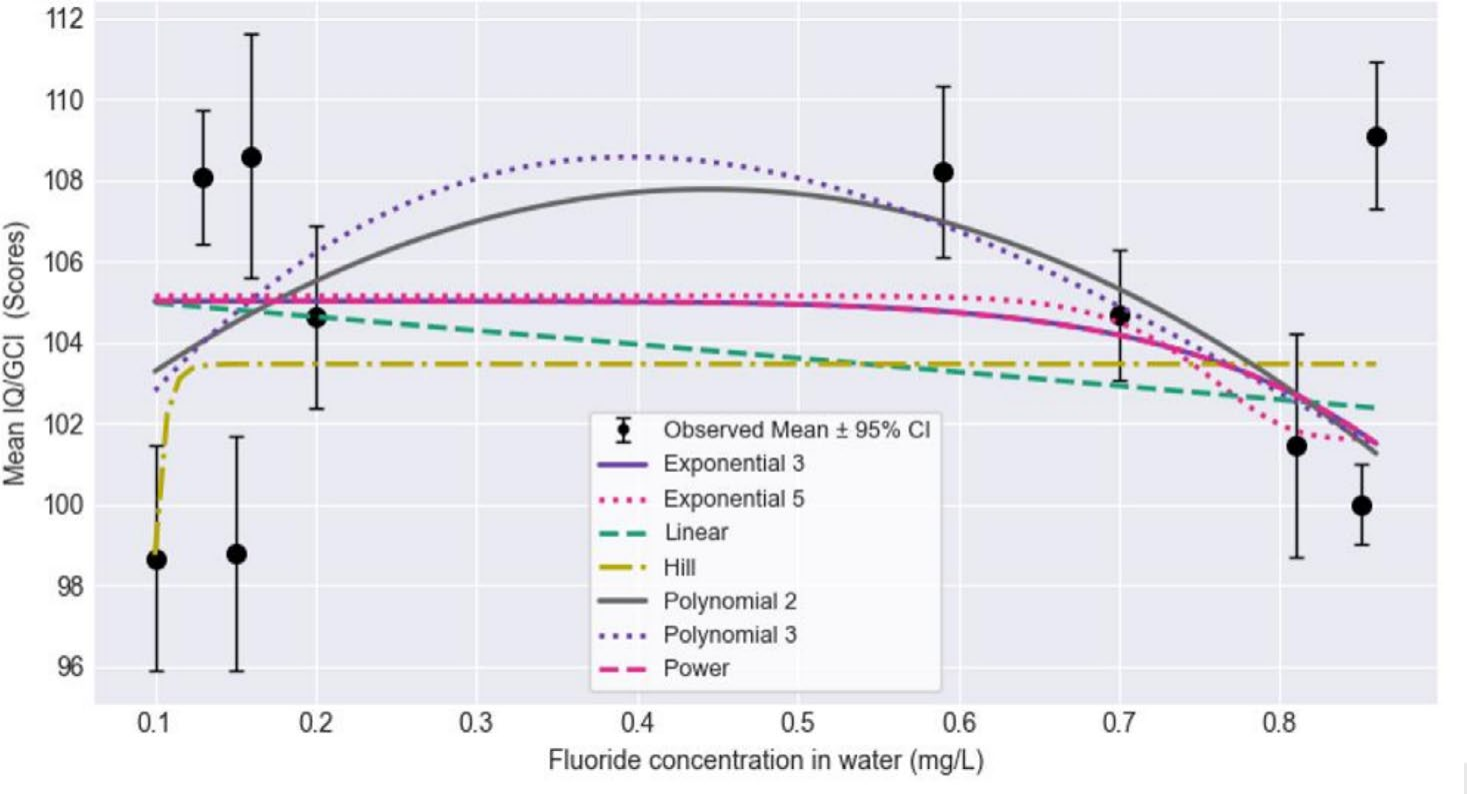
Benchmark dose models for fluoride concentration in water and cognitive function outcomes from studies in fluoridated communities.

These concentration‐response models were also unusable or questionable. A visual inspection of the mean IQ/GCI summary data (Figure 3) did not reveal a functional relationship between water F concentration and intelligence outcome in this exposure range (Figure SE). The model fit was poor (*p*‐value for Goodness of Fit < 0.1).

## 4 Discussion

A review of seven studies conducted in six countries relevant to community water fluoridation revealed a wide variation in sample characteristics and cognition scores. Furthermore, there are substantial differences in sample selection, data collection, the sources of fluoride, and the method used for adjusting urine dilution. A meta‐analysis showed that the pooled effect size of the association between MUF and cognition scores is not statistically significant. In addition, all water fluoridation studies consistently showed a positive effect, thus weakening the argument for selecting IQ as a clinical endpoint for assessing the potential risk posed by fluoride in this exposure range.

The maternal urinary studies for the concentration‐response analysis did not meet the EPA guidance criteria for acceptable quality (Table S1) and for combining the data from different studies. The analysis of water fluoridation concentration and cognition studies is consistent with the results of the maternal urinary fluoride studies. In both analyses, modeling the data to fit a concentration‐response curve showed no functional relationship. In addition, the data quality assessment showed serious flaws that make these maternal urinary epidemiological studies unacceptable for hazard assessment. The MUF‐IQ response profile does not present a homogeneous picture of the concentration‐response relationship. The results are not generalizable beyond these datasets. The BMC and BMCL cannot be reliably estimated because at least two studies employed non‐probability cluster sampling without adjusting the standard error for the cluster design effect. For these reasons, a concentration‐response analysis for calculating BMC and BMCL is not appropriate.

In contrast, the EPA based its current BMDL of 1.87 mg/L F on a dataset from a single U.S. study conducted by Dean [2, 24, 25]. This dataset was deemed sufficiently large and robust to support statistical analysis, and the researchers applied objective criteria for severe dental fluorosis; the dichotomous Hill model adequately fits the data (Figure SF). Spencer et al. have discussed the implications of this BMDL for determining the fluoride reference dose to achieve an optimal fluoride level for preventing tooth decay [25].

The present analysis differed from that of Grandjean et al. [11] in several ways. First, the sample mean IQ/GCI score and fluoride concentration summary data at the group level were used, rather than the regression coefficient summary data derived from spot urine samples. This approach is similar to that used by the EPA to establish the current BMD and BMDL for fluoride concentration in water [2]. Due to the short half‐life of fluoride (< 6 h) and significant within and between variations in urinary flow and creatinine excretion rates, multiple, standardised individual spot or 24‐h urine samples are necessary to produce an accurate, precise, and reliable measure of long‐term fetal fluoride exposure that can ensure the validity of study findings [26, 27, 28, 29]. Furthermore, the EPA considers a single spot urine sample unacceptable for risk assessment [17]. However, the group‐level fluoride exposure measure during pregnancy may be adequate for exploratory analysis. Weisskopf and Webster argued that a group‐level measure may be preferable when valid measures are unavailable at the individual level [30]. Second, Grandjean et al. [11] used a coefficient of −0.94 for a 1 mg/L urinary F increase to demonstrate homogeneity in the dose response. This analysis is problematic when the description in their paper shows positive coefficients (log‐transformed 0.08 or 0.18) for their study population. Third, Grandjean et al. [11] excluded the INMA cohort without an explanation. All four studies have similar designs and similar limitations [6, 28]. Fourth, although an analysis using the adjusted regression coefficient may appear superior, the study authors' selection of variables was based on a prediction model rather than a causal inference model. Not all studies used the same variables for statistical adjustments. Fifth, mixing estimates derived from Generalised Estimating Equation (ELEMENT) and fixed effects models introduces model heterogeneity, which can distort the estimated BMC and BMCL values. Finally, a BMR of 0.5 STD was used as recommended by the EPA instead of a 1 IQ point change in response. The data structure did not allow the use of models with a 1 IQ point BMR. Considering the 10‐point IQ difference between the OCC and MIREC lowest exposure groups, the limited number of studies, and the fact that the surrogate MUF exposure differs from blood lead measurement in quantifying lead‐related IQ deficits, these findings do not support the selection of a 1 IQ point change in response [31].

For several reasons, the ELEMENT cohort should not be combined with other studies to derive a concentration‐response analysis. First, the EPA does not regulate the exposure source salt. Salt is also a potential confounder that Goodman et al. [13] did not address [32]. Second, the study authors have raised concerns about the validity of spot MUF exposure as a long‐term measure of fetal fluoride exposure in this study [13, 33]. Third, the subgroup differences are statistically significant, and the effect size is characterized by high heterogeneity. Finally, there may be systematic differences in reported and unreported results [34]. For example, while Thomas [34], in her unpublished thesis, found that concurrent urinary fluoride exposure showed a positive association with WASI scores (*β* = 1.32 per 1 mg/L F increase), Bashash et al. [21] reported a 0.89 lower IQ (95% CI: −2.63, 0.85) per 0.5 mg/L F increase.

## 5 Strengths and Limitations

While this analysis's strengths include using a meta‐analysis to synthesize the effect sizes from all cohort studies and following the EPA guidance, it also has several limitations. The cognition scores in different studies are not directly comparable. The cognition assessment requires calibration that was not standardized across multiple studies. The data quality, the limited number of studies, non‐probability sampling, unadjusted IQ scores, analysis based on secondary data, and the range of exposure should be considered when applying the findings from these studies to inform the development of regulatory actions. Notwithstanding the limitations of the fluoride‐IQ studies, the lack of association in the pooled SMD estimate and the pooled regression coefficient estimates associated with MUF studies, as well as the positive association in multivariate analysis in three water fluoridation studies, provide assurance of safety against any potential harm associated with fluoridated water [18, 19, 20].

## 6 Conclusion

The maternal urinary fluoride datasets did not show a homogeneous response, and the neurodevelopmental hazard has not been adequately demonstrated to warrant proceeding to the next steps of risk assessment. Recent studies from Sweden, China, Canada, Denmark and Australia have not shown deficits in cognitive scores at low levels of fluoride exposure [12, 18, 20, 35, 36, 37, 38]. The IQ scores did not improve after the cessation of water fluoridation in Calgary, whereas there was a detrimental effect on dental caries outcomes [39, 40]. The public can be reassured that the fluoride exposure range examined here, consistent with community water fluoridation, did not affect cognitive function. More appropriate studies in endemic fluorosis areas are needed to better understand if fluoride causes adverse cognitive outcomes in that population.

## Author Contributions

Jayanth V. Kumar conceptualised and designed the study, supervised the overall project, conducted the literature review, acquired the data, performed the meta‐analysis, and led the drafting of the manuscript. Mark E. Moss conceptualised and designed the study, performed the literature review, reviewed the meta‐analysis, led the risk of bias assessment, and revised the manuscript for important intellectual content. Honghu Liu served as the chief statistician, reviewing statistical analysis and critically revising the manuscript for its intellectual content. Susan Fisher‐Owens critically reviewed the literature and the manuscript for its intellectual content and revised the manuscript accordingly. Andrew Rugg‐Gunn critically reviewed the literature on urinary fluoride studies and the manuscript for its intellectual content. Julia Kuring critically reviewed the literature on psychometric tests used in fluoride‐IQ studies and their relevance for choosing the benchmark response. All authors approved the final manuscript as submitted and agree to be accountable for all aspects of the work.

## Ethics Statement

This project did not involve human data or participants and utilised publicly available data; therefore, an IRB assessment was not necessary, as per the institutional policy of the affiliated organisations.

## Conflicts of Interest

Jayanth V. Kumar and Mark E. Moss are members of the American Dental Association's National Fluoridation Advisory Committee. Jayanth V. Kumar was a reviewer of the National Academies of Sciences, Engineering, and Medicine report, *Review of the Revised NTP Monograph on the Systematic Review of Fluoride Exposure and Neurodevelopmental and Cognitive Health Effects*: *A Letter Report* (*2021*). Susan Fisher‐Owens is a member of the American Academy of Paediatrics' Section on Oral Health. She was a co‐author of ‘Fluoride Use in Caries Prevention in the Primary Care Setting’ and ‘Review of Safety, Frequency and Intervals of Preventive Fluoride Varnish Application for Children.’ She consults for Arcora Foundation on medical dental integration and has research funding for medical dental integration from Health Resources Services Administration (HRSA) D88HP37553. She serves on an independent DSMB for a study funded by Colgate. Andrew Rugg‐Gunn is a member of the British Fluoridation Society. Jayanth V. Kumar contributed to this article in his personal capacity. The views expressed are his own and do not necessarily represent the views or opinions of the National Institutes of Dental and Craniofacial Research, NIH, or the California Department of Public Health.

## Supporting information


**Data S1:** cdoe70027‐sup‐0001‐TableS1.pdf.

## Data Availability

The data underlying this article are available in the article and in its online Supporting Information.
