# Supplementary material for: Fluoride and Neurodevelopmental Hazard Modelling: An Assessment of Concentration‐Response Analysis
Source: Community Dent Oral Epidemiol. 2025 Oct 31;54(1):101–11. doi: 10.1111/cdoe.70027 (PMC12808860; doi:10.1111/cdoe.70027)
Supplement: Supplementary file 1 — Data S1: cdoe70027‐sup‐0001‐TableS1.pdf. [file CDOE-54-101-s001.pdf]

Supplementary Materials

1. EPA Technical Guide
2. Population-Intervention-Comparison-Outcome-Time-Setting Statement
3. Supplementary Figure A - Study Selection Flow Diagram
4. Supplementary Figure B - OCC data extraction and analysis
5. Supplementary Figure C - Forest Plot of MUF meta-analyses
6. Supplementary Figure D - MUF and IQ/GCI Benchmark Concentration Analysis
7. Supplementary Figure E - Water F and IQ Benchmark Concentration Analysis
8. Supplementary Figure F - Water F and Severe Dental Fluorosis Benchmark Concentration Analysis
9. Supplementary Table S1 - Data Quality Criteria and Assessment

EPA Technical Guide for Benchmark Dose Modeling

Source: U.S. EPA. Benchmark Dose (BMDS) Technical Guidance Document (Final, 2012). U.S. Environmental Protection Agency, Washington, DC, EPA/100/R-12/001.

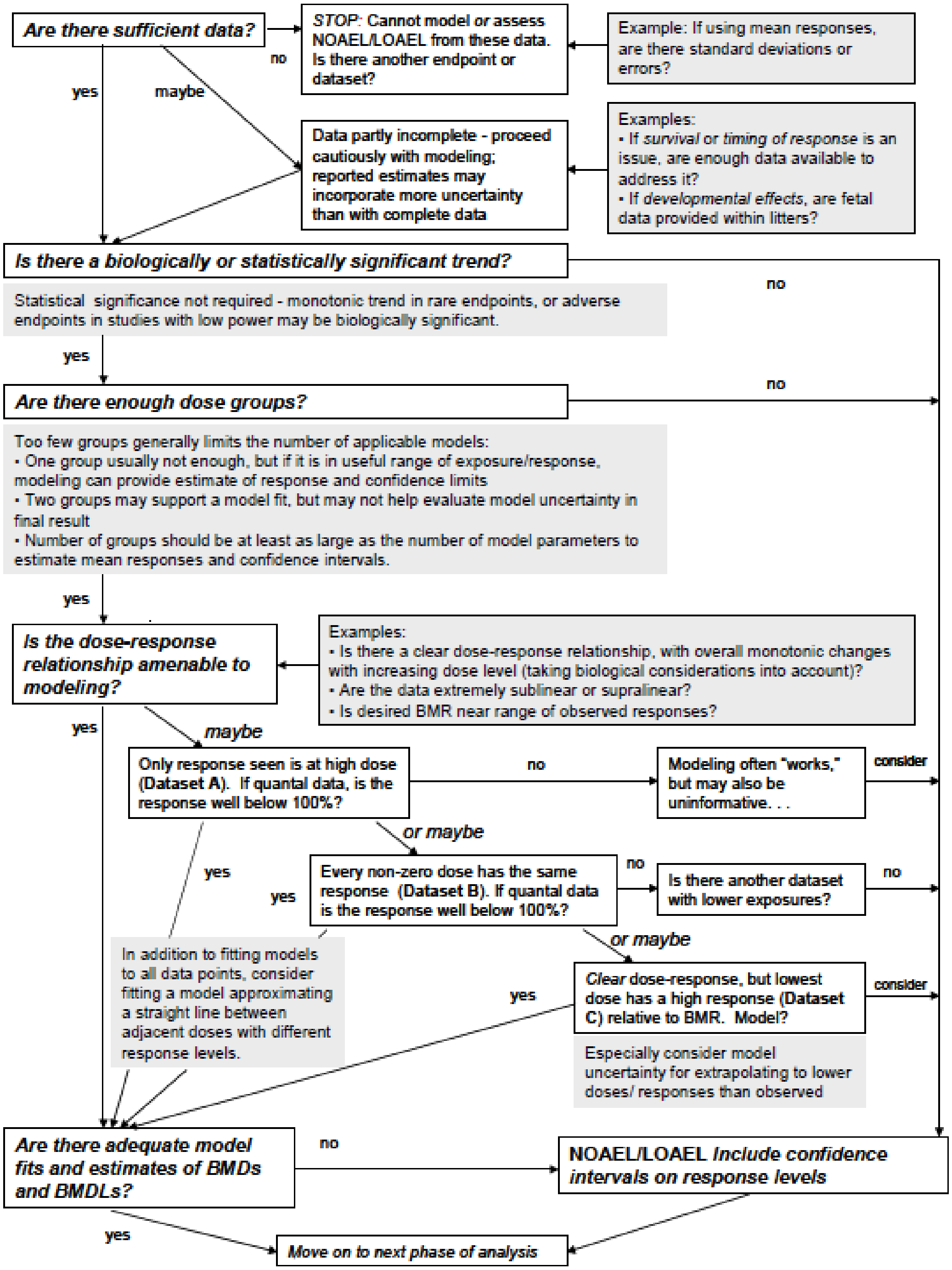

Figure 2A. Flowchart of data evaluation steps for determining BMD modeling feasibility. (See Figure 2B for Datasets A, B, and C.)

EPA Technical Guide for Benchmark Dose Modeling

Source: U.S. EPA. Benchmark Dose (BMDS) Technical Guidance Document (Final, 2012). U.S. Environmental Protection Agency, Washington, DC, EPA/100/R-12/001.

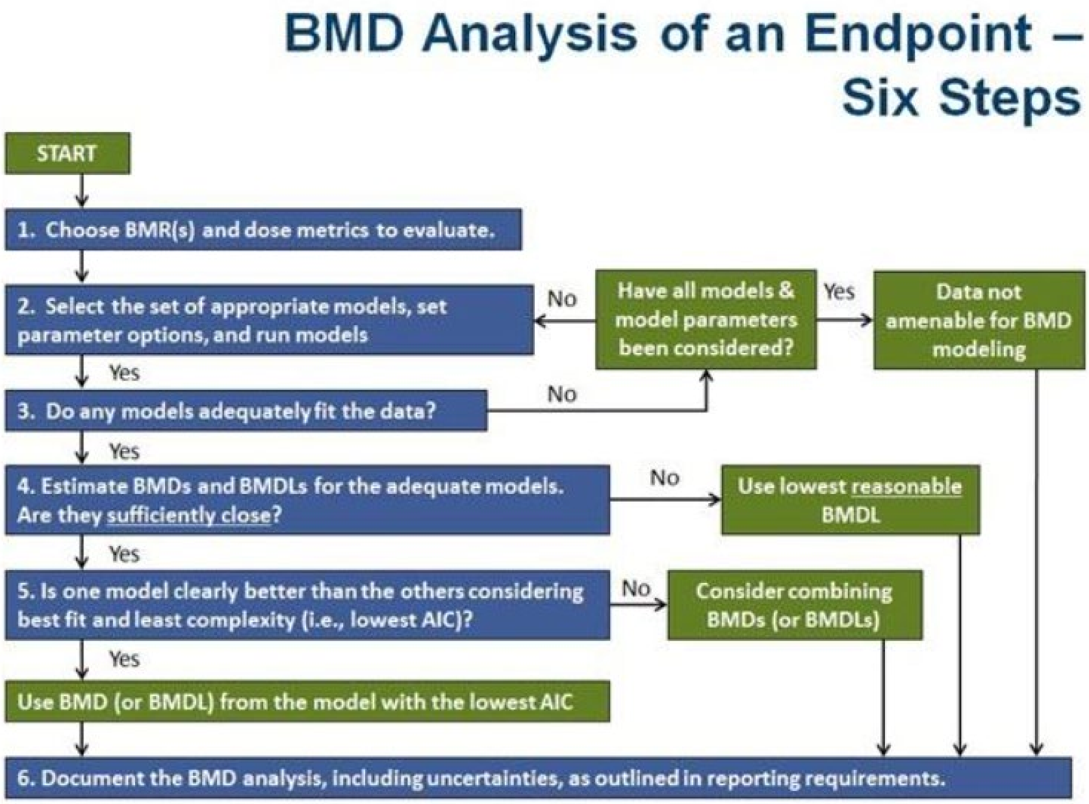

Population-Intervention-Comparison-Outcome-Time-Setting Statement

Study population	Children and Pregnant Women
Intervention/exposure	Higher fluoride assessed in drinking water or urine samples (child and maternal).
Comparison	Similar group(s) to exposure group but with lower fluoride exposure
Outcomes	Children's cognition or IQ scores
Time	Studies published through December 2024
Setting	Children from fluoridated and non-fluoridated areas
Study design	Dose-response modeling to assess viability for revising safety threshold of fluoride in drinking water

Figure A. Flow diagram of the publications selected for analyses

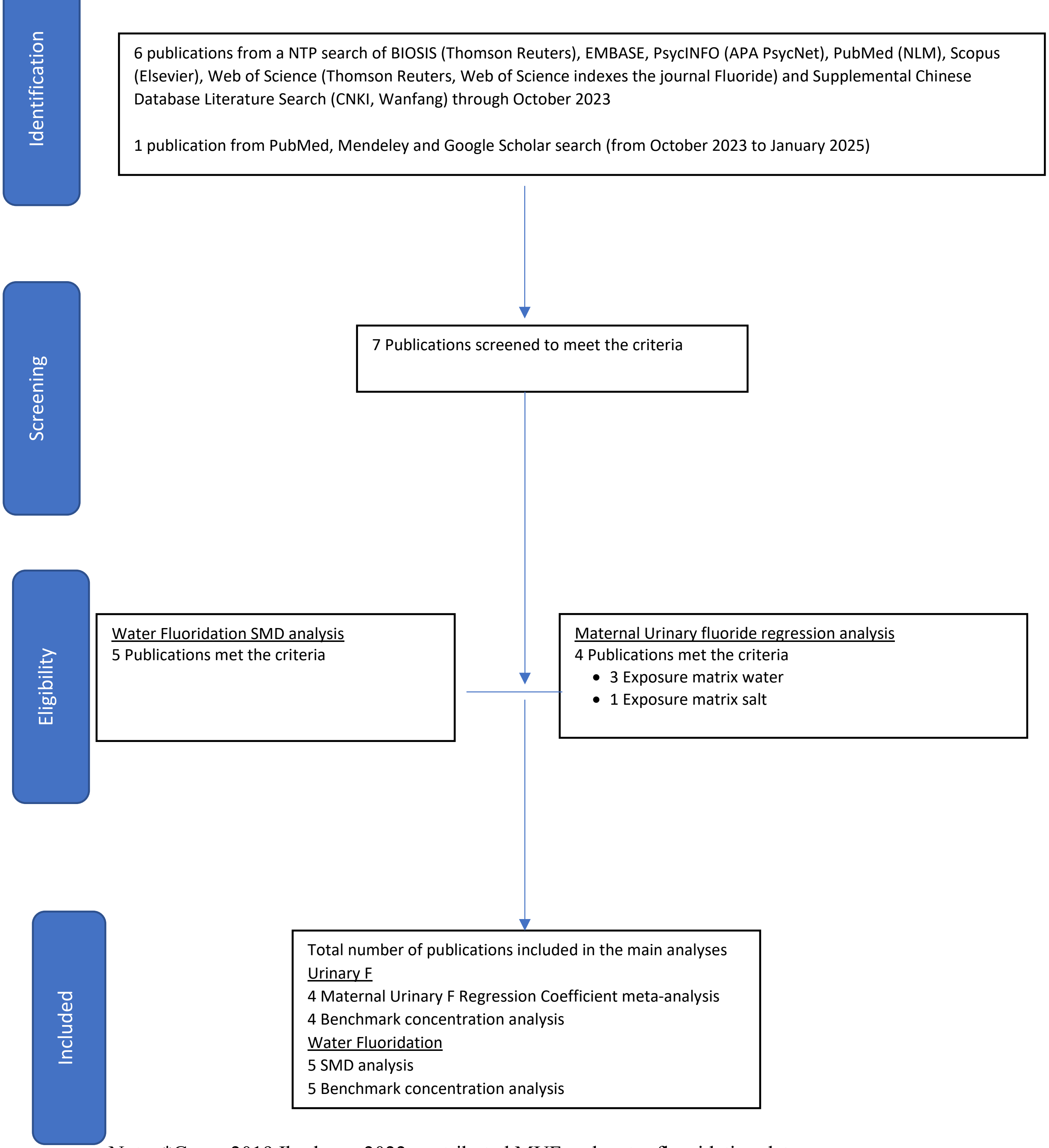

Note: *Green 2019 Ibarluzea 2022 contributed MUF and water fluoridation data.

Supplementary Figure B -

Results of data extracted from Figure 2 Grandjean et al.

Descriptive

Descriptives

	FSIQ	MUF
N	803	803
Missing	0	0
Mean	98.9	0.586
Median	98.0	0.520
Standard deviation	12.8	0.323
Minimum	57.8	0.0800
Maximum	137	3.05

Descriptives

Descriptives

	MUF - Transform 2	MUF	FSIQ
N	1	408	408
	2	395	395
Missing	1	0	0
	2	0	0
Mean	1	0.368	98.1
	2	0.812	99.8
Median	1	0.380	97.7
	2	0.710	98.2
Standard deviation	1	0.0944	13.3
	2	0.321	12.2
Minimum	1	0.0800	57.8
	2	0.530	60.7
Maximum	1	0.520	137
	2	3.05	134

General Linear Model

Analysis plot

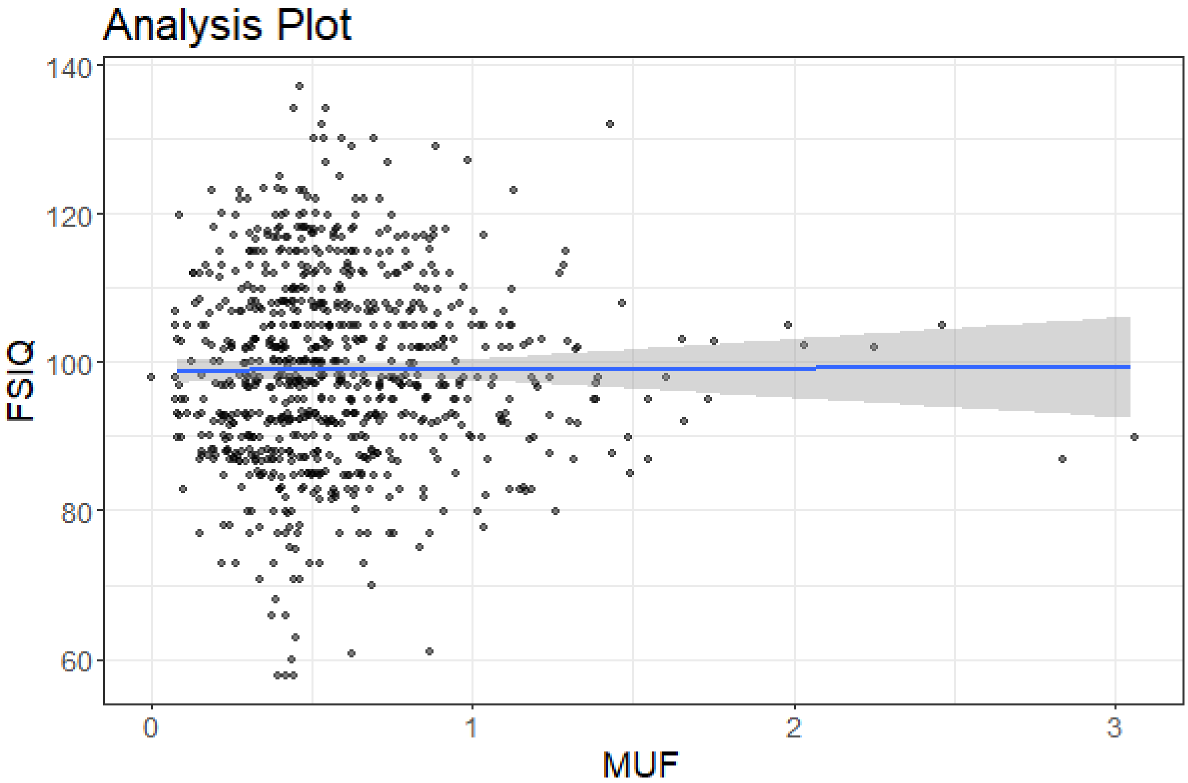

Estimates and effect sizes for categorical predictors

Variable	Level	Estimate	95% Confidence Interval Lower	95% Confidence Interval Upper
Numeric Variables (Estimates reported are slopes/intercepts)				
Intercept		98.844	97.01	100.68
Slope: MUF		0.153	-2.59	2.9

R^2 and semi-partial R^2 estimates

Source	Estimate
model	0.00000
MUF	1.49e-5
Correlation coefficient	0.00386

Independent Samples T-Test

Independent Samples T-Test

		Statistic	df	p	Mean difference	SE difference	95% Confidence Interval Lower	95% Confidence Interval Upper
FSIQ	Student's t	-1.94	801	0.053	-1.75	0.902	-3.52	0.0227

Note. H_a $\mu_1 \neq \mu_2$

Group Descriptives

	Group	N	Mean	Median	SD	SE
FSIQ	1	408	98.1	97.7	13.3	0.657
	2	395	99.8	98.2	12.2	0.616

Group 1 - Lower F ; 2 - Higher F

Plots

FSIQ

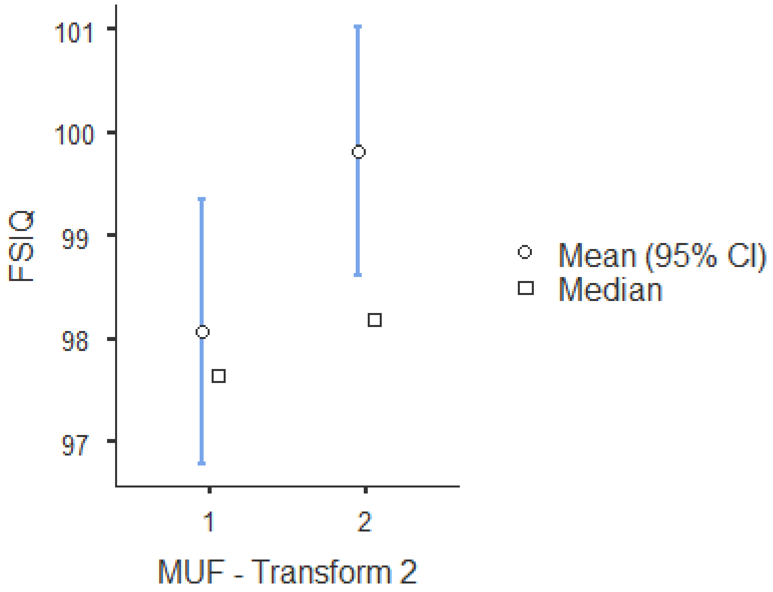

References

[1] The jamovi project (2023). *jamovi*. (Version 2.4) [Computer Software]. Retrieved from https://www.jamovi.org.

[2] R Core Team (2022). *R: A Language and environment for statistical computing*. (Version 4.1) [Computer software]. Retrieved from https://cran.r-project.org. (R packages retrieved from CRAN snapshot 2023-04-07).

Supplementary Figure C

Source for Table 2. Meta-analysis for maternal urinary fluoride (MUF) effects by replacing the negative OCC regression coefficient.

Fixed or random effects analysis of regression coefficients associated with 1 mg/L increase in spot maternal urinary F.

Reproduced Grandjean et al. analysis Table S2 with the OCC negative coefficent -Fixed Effects

Study or Subgroup	Regression Coefficient	SE	Weight	Regression Coefficient IV, Fixed, 95% CI
**2.1.1 Water Fluoridation**				
Green 2019	-2.01	1.43	30.5%	-2.01 [-4.81, 0.79]
Ibarluzea 2021	3.37	2.786	0.0%	3.37 [-2.09, 8.83]
**Subtotal (95% CI)**			**30.5%**	**-2.01 [-4.81, 0.79]**
Heterogeneity: Not applicable				
Test for overall effect: Z = 1.41 (P = 0.16)				
**2.1.2 Salt Fluoridation**				
Goodman 2022	-4.09	1.57	25.3%	-4.09 [-7.17, -1.01]
**Subtotal (95% CI)**			**25.3%**	**-4.09 [-7.17, -1.01]**
Heterogeneity: Not applicable				
Test for overall effect: Z = 2.61 (P = 0.009)				
**2.1.3 Naturally Occurring Fluoride**				
Grandjean 2023	-0.94	1.19	44.1%	-0.94 [-3.27, 1.39]
**Subtotal (95% CI)**			**44.1%**	**-0.94 [-3.27, 1.39]**
Heterogeneity: Not applicable				
Test for overall effect: Z = 0.79 (P = 0.43)				
**Total (95% CI)**			**100.0%**	**-2.07 [-3.61, -0.52]**
Heterogeneity: Chi² = 2.56, df = 2 (P = 0.28); I² = 22%				
Test for overall effect: Z = 2.61 (P = 0.009)				
Test for subgroup differences: Chi² = 2.56, df = 2 (P = 0.28), I² = 21.8%				

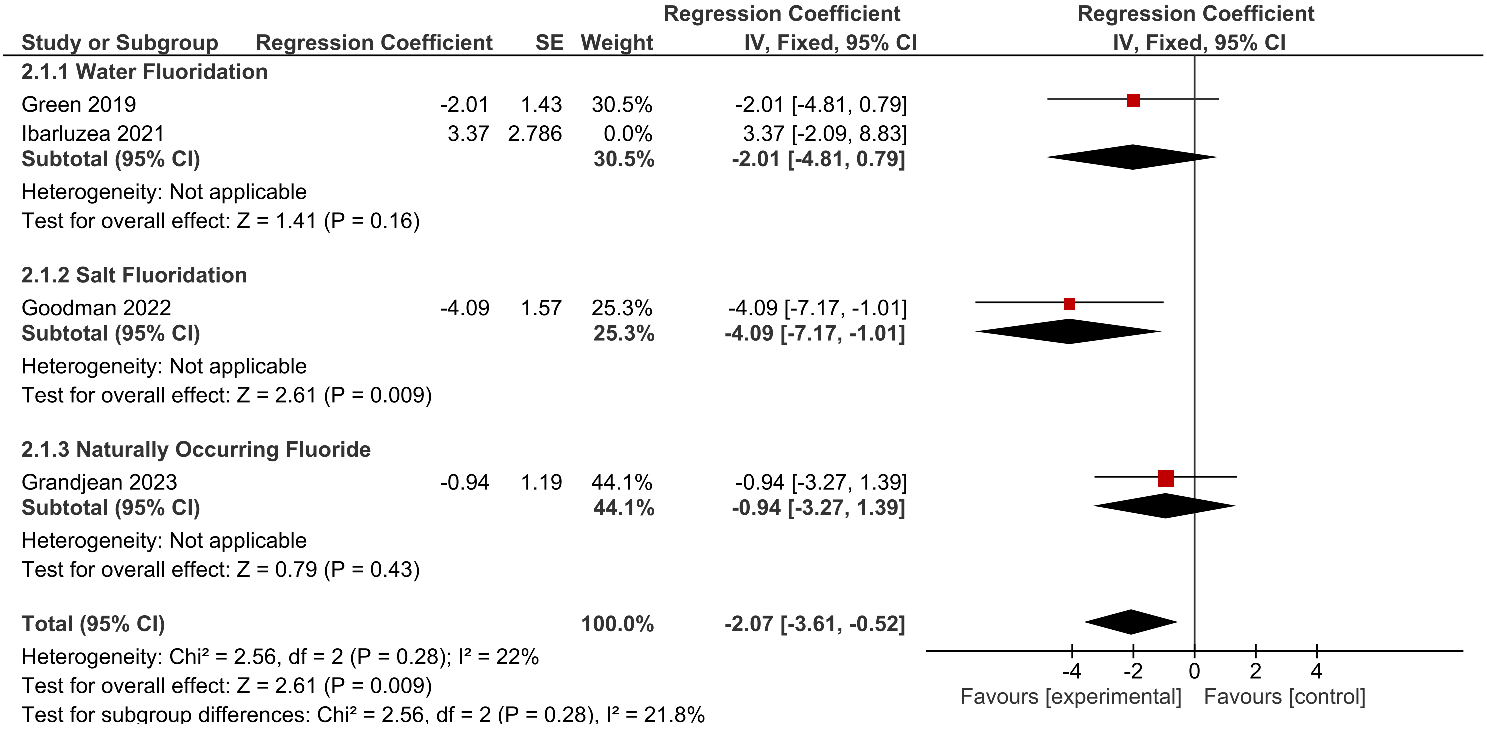

Includes MIREC, ELEMENT cohorts, and OCC positive coefficient

Study or Subgroup	Regression Coefficient	SE	Weight	Regression Coefficient IV, Random, 95% CI
**1.1.1 Water Fluoridation**				
Green 2019	-2.01	1.43	32.4%	-2.01 [-4.81, 0.79]
Ibarluzea 2021	3.37	2.786	0.0%	3.37 [-2.09, 8.83]
**Subtotal (95% CI)**			**32.4%**	**-2.01 [-4.81, 0.79]**
Heterogeneity: Not applicable				
Test for overall effect: Z = 1.41 (P = 0.16)				
**1.1.2 Salt Fluoridation**				
Goodman 2022	-4.09	1.57	29.9%	-4.09 [-7.17, -1.01]
**Subtotal (95% CI)**			**29.9%**	**-4.09 [-7.17, -1.01]**
Heterogeneity: Not applicable				
Test for overall effect: Z = 2.61 (P = 0.009)				
**1.1.3 Naturally Occurring Fluoride**				
Grandjean 2023	0.26	1.16	37.7%	0.26 [-2.01, 2.53]
**Subtotal (95% CI)**			**37.7%**	**0.26 [-2.01, 2.53]**
Heterogeneity: Not applicable				
Test for overall effect: Z = 0.22 (P = 0.82)				
**Total (95% CI)**			**100.0%**	**-1.78 [-4.28, 0.73]**

Heterogeneity: Tau² = 2.99; Chi² = 5.15, df = 2 (P = 0.08); I² = 61%

Test for overall effect: Z = 1.39 (P = 0.16)

Test for subgroup differences: Chi² = 5.15, df = 2 (P = 0.08), I² = 61.2%

Regression Coefficient
IV, Random, 95% CI

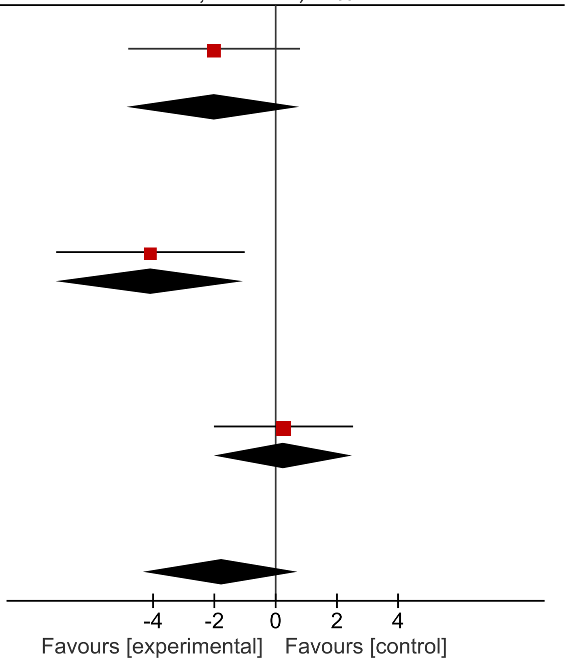

Includes all 4 cohorts with OCC positive coefficient

Study or Subgroup	Regression Coefficient	SE	Weight	Regression Coefficient IV, Random, 95% CI
**1.1.1 Water Fluoridation**				
Green 2019	-2.01	1.43	27.9%	-2.01 [-4.81, 0.79]
Ibarluzea 2021	3.37	2.786	14.5%	3.37 [-2.09, 8.83]
**Subtotal (95% CI)**			**42.4%**	**0.15 [-5.02, 5.32]**
Heterogeneity: Tau² = 9.57; Chi² = 2.95, df = 1 (P = 0.09); I² = 66%				
Test for overall effect: Z = 0.06 (P = 0.96)				
**1.1.2 Salt Fluoridation**				
Goodman 2022	-4.09	1.57	26.1%	-4.09 [-7.17, -1.01]
**Subtotal (95% CI)**			**26.1%**	**-4.09 [-7.17, -1.01]**
Heterogeneity: Not applicable				
Test for overall effect: Z = 2.61 (P = 0.009)				
**1.1.3 Naturally Occurring Fluoride**				
Grandjean 2023	0.26	1.16	31.5%	0.26 [-2.01, 2.53]
**Subtotal (95% CI)**			**31.5%**	**0.26 [-2.01, 2.53]**
Heterogeneity: Not applicable				
Test for overall effect: Z = 0.22 (P = 0.82)				
**Total (95% CI)**			**100.0%**	**-1.06 [-3.63, 1.50]**
Heterogeneity: Tau² = 4.09; Chi² = 7.98, df = 3 (P = 0.05); I² = 62%				
Test for overall effect: Z = 0.81 (P = 0.42)				
Test for subgroup differences: Chi² = 5.23, df = 2 (P = 0.07), I² = 61.7%				

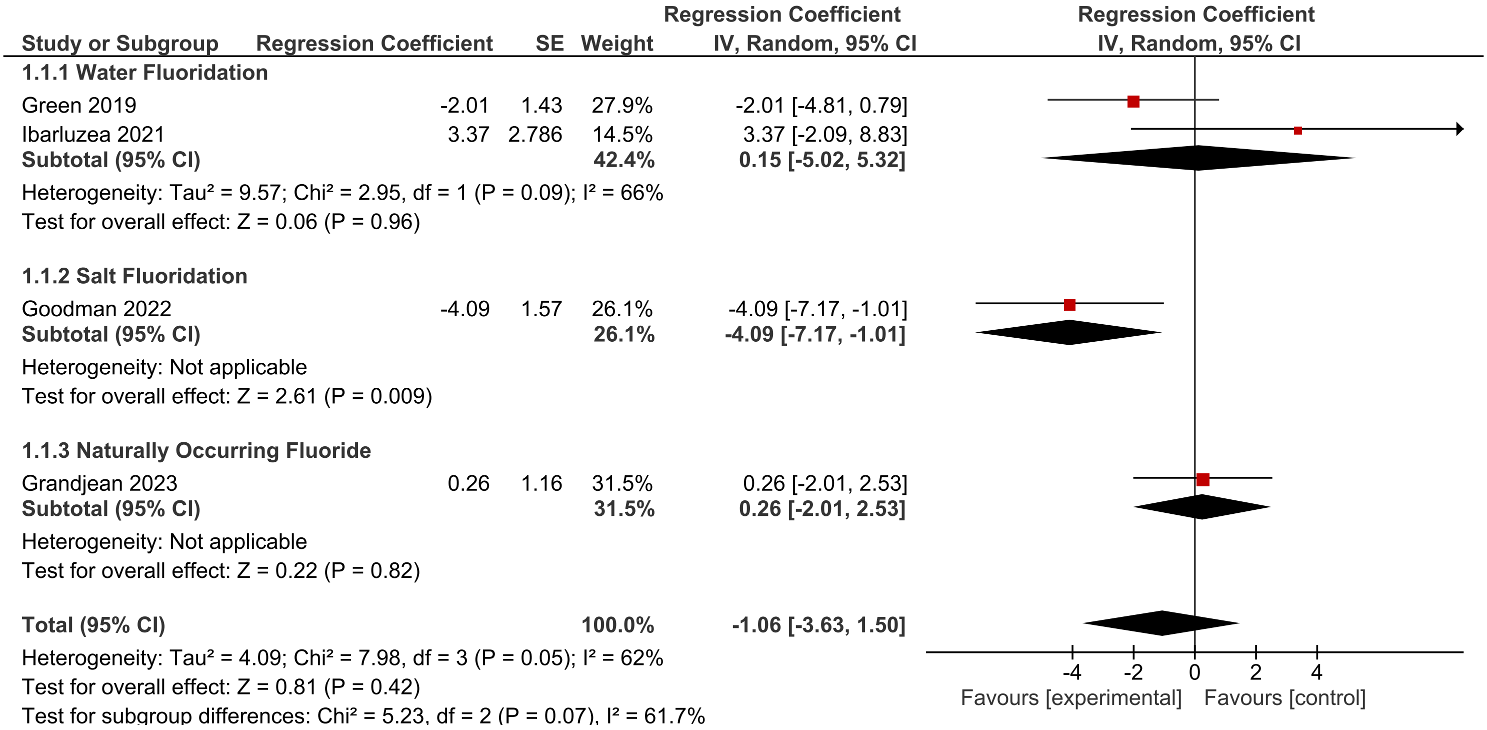

Excluding the ELEMENT cohort (Salt fluoridation)

Study or Subgroup	Regression Coefficient	SE	Weight	Regression Coefficient IV, Random, 95% CI
**1.1.1 Water Fluoridation**				
Green 2019	-2.01	1.43	38.1%	-2.01 [-4.81, 0.79]
Ibarluzea 2021	3.37	2.786	15.4%	3.37 [-2.09, 8.83]
**Subtotal (95% CI)**			**53.5%**	**0.15 [-5.02, 5.32]**
Heterogeneity: Tau² = 9.57; Chi² = 2.95, df = 1 (P = 0.09); I² = 66%				
Test for overall effect: Z = 0.06 (P = 0.96)				
**1.1.2 Salt Fluoridation**				
Goodman 2022	-4.09	1.57	0.0%	-4.09 [-7.17, -1.01]
**Subtotal (95% CI)**				**Not estimable**
Heterogeneity: Not applicable				
Test for overall effect: Not applicable				
**1.1.3 Naturally Occurring Fluoride**				
Grandjean 2023	0.26	1.16	46.5%	0.26 [-2.01, 2.53]
**Subtotal (95% CI)**			**46.5%**	**0.26 [-2.01, 2.53]**
Heterogeneity: Not applicable				
Test for overall effect: Z = 0.22 (P = 0.82)				
**Total (95% CI)**			**100.0%**	**-0.13 [-2.51, 2.25]**
Heterogeneity: Tau² = 1.82; Chi² = 3.40, df = 2 (P = 0.18); I² = 41%				
Test for overall effect: Z = 0.10 (P = 0.92)				
Test for subgroup differences: Chi² = 0.00, df = 1 (P = 0.97), I² = 0%				

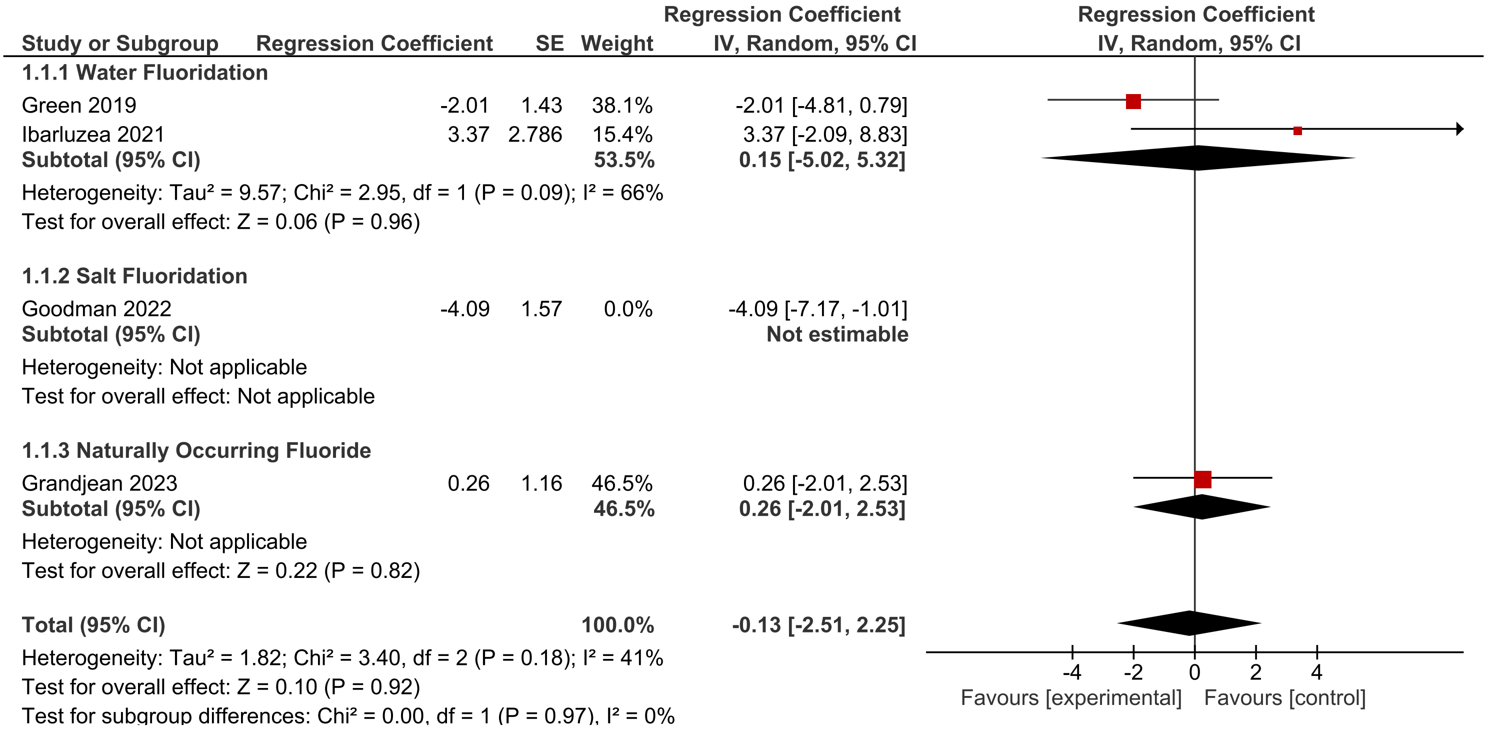

Leave-one-out Meta-analysis

Continuous Random-Effects Model

Studies	Estimate	Lower bound	Upper bound	Std. error	p-Val
Overall	-1.06	-3.63	1.50	1.31	0.42
- Green	-0.57	-4.33	3.19	1.92	0.77
- Ibarluzea	-1.78	-4.28	0.73	1.28	0.16
- Goodman	-0.13	-2.51	2.25	1.21	0.92
- Grandjean	-1.57	-4.99	1.86	1.75	0.37

Leave-one-out Forest Plot

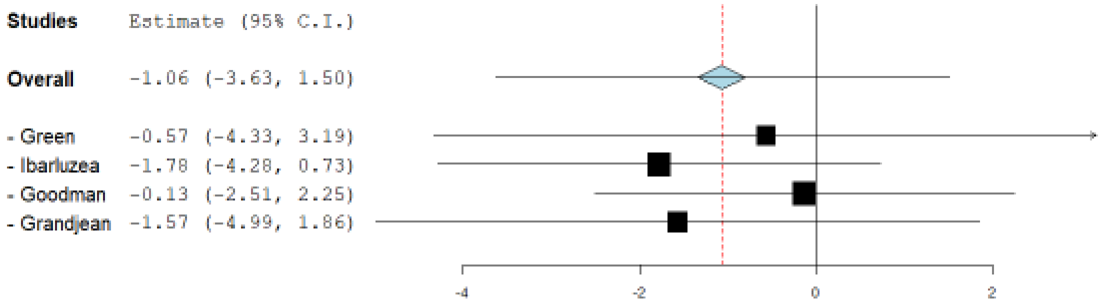

Supplementary Figure D- Benchmark Dose Analysis

Session for MUF and IQ/GCI Includes ELEMENT

Includes Maternal Urinary Fluoride studies: ELEMENT, MIREC, INMA, OCC.

Dataset

Name: MUF and IQ/GCI Includes ELEMENT

**Dose (mg/L)**	0.37	0.4	0.45	0.54	0.69	0.81	0.82	1.01
**N**	408	238	123	77	162	395	124	112
**Mean ± SD (Score)**	98.1 ± 13.3	108.07 ± 13.31	98.67 ± 15.7	95.37 ± 10.31	108.21 ± 13.72	99.8 ± 12.2	101.47 ± 15.5	96.8 ± 11.16

Test 1 Dose Response: 0
Test 2 Homogeneity of Variance: <0.0001
Test 3 Variance Model Selection: <0.0001

Settings

**Setting**	**Value**
**BMR**	0.5 Standard Deviation
**Distribution**	Normal + Constant variance
**Adverse Direction**	Down (↓)
**Maximum Polynomial Degree**	3
**Confidence Level (one sided)**	0.95

Maximum Likelihood Approach

**Model**	**BMDL**	**BMD**	**BMDU**	***P*-Value**	**AIC**	**Scaled Residual at Control**	**Scaled Residual near BMD**	**Recommendation and Notes**
Exponential 3	1.035	1.047	1.11	0	13259.155	-5.123	0.134	**Questionable** Residual at control > 2.0 Goodness of fit p-value < 0.1 Constant variance test failed (Test 2 p-value < 0.05) BMDL/highest dose ratio > 1.0 BMD/highest dose ratio > 1.0
Exponential 5	1.008	1.047	1.11	0	13261.155	-5.123	0.134	**Questionable** Residual at control > 2.0 Goodness of fit p-value < 0.1 Constant variance test failed (Test 2 p-value < 0.05) BMD/highest dose ratio > 1.0

Linear	1.449	3.158	-	0	13268.168	-5.038	-2.562	**Questionable**<br>|Residual near BMD| > 2.0<br>Residual at control > 2.0<br>Goodness of fit p-value < 0.1<br>Constant variance test failed (Test 2 p-value < 0.05)<br>BMDL/highest dose ratio > 1.0<br>BMD/highest dose ratio > 1.0
Hill	-	-	-	0	13260.567	-1.412	-	**Unusable**<br>Did not successfully execute.
Polynomial 2	0.351	0.358	0.781	0	13254.842	-4.47	-4.47	**Questionable**<br>|Residual near BMD| > 2.0<br>Residual at control > 2.0<br>Goodness of fit p-value < 0.1<br>Constant variance test failed (Test 2 p-value < 0.05)
Polynomial 3	0.022	0.218	0.973	0	13252.691	-3.759	-3.759	**Questionable**<br>|Residual near BMD| > 2.0<br>lowest dose/BMDL ratio > 3.0<br>lowest dose/BMDL ratio > 10.0<br>Residual at control > 2.0<br>Goodness of fit p-value < 0.1<br>Constant variance test failed (Test 2 p-value < 0.05)<br>BMD/BMDL ratio > 3.0
Power	1.043	1.046	1.049	0	13259.173	-5.12	0.13	**Questionable**<br>Residual at control > 2.0<br>Goodness of fit p-value < 0.1<br>Constant variance test failed (Test 2 p-value < 0.05)<br>BMDL/highest dose ratio > 1.0<br>BMD/highest dose ratio > 1.0

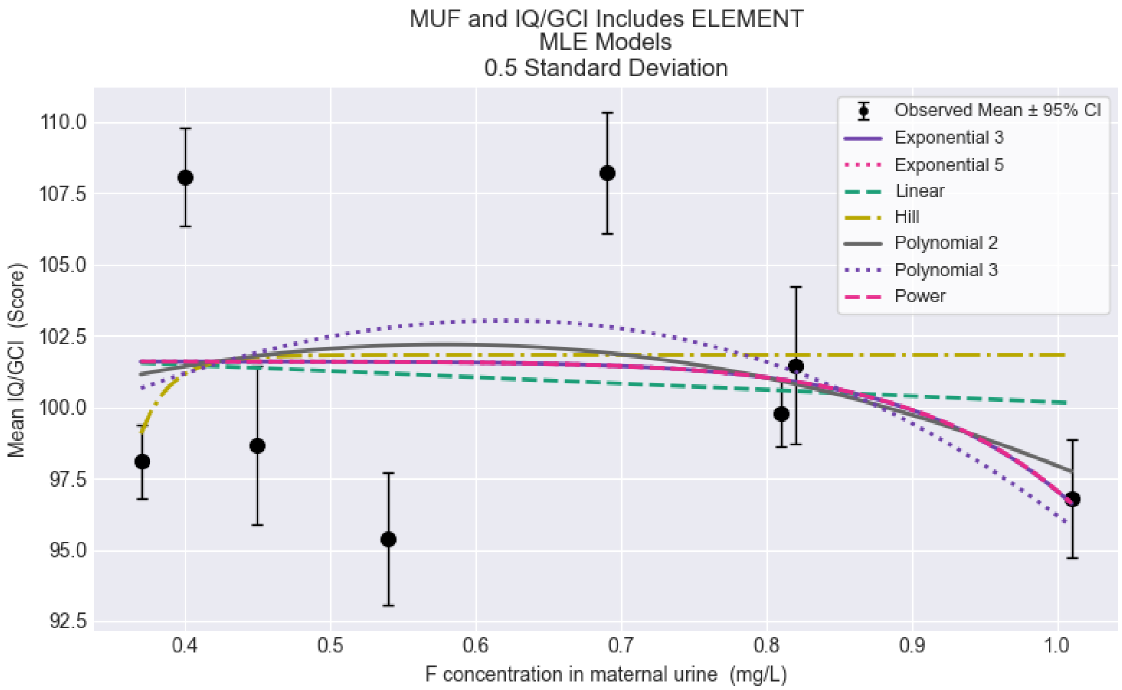

Individual Model Results

Linear Model

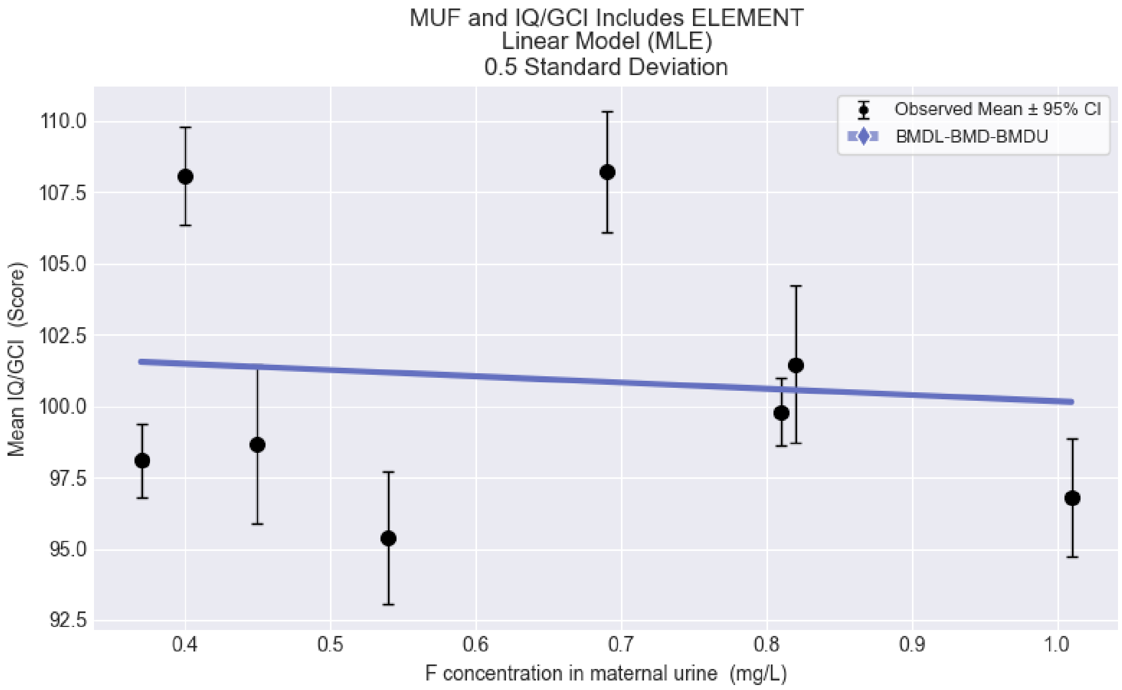


```
        Linear Model
═══════════════════════════
```

Version: pybmds 24.1 (bmdscore 24.1)

Input Summary:

BMR	0.5 Standard Deviation
Distribution	Normal + Constant variance
Modeling Direction	Down (↓)
Confidence Level (one sided)	0.95
Modeling Approach	MLE
Degree	1

Parameter Settings:

Parameter	Initial	Min	Max
g	0	-1e+06	1e+06
b1	0	-1e+06	1e+06
alpha	0	-18	18

Modeling Summary:

BMD	3.15837
BMDL	1.44934
BMDU	-9999
AIC	13268.2
Log-Likelihood	-6631.08
P-Value	0
Model d.f.	6

Model Parameters:

Variable	Estimate	On Bound	Std Error
g	102.359	no	0.99796
b1	-2.18949	no	1.55295
alpha	191.281	no	1278.11

Goodness of Fit:

Dose	N	Sample Mean	Model Fitted Mean	Scaled Residual
0.37	408	98.1	101.549	-5.03757
0.4	238	108.07	101.484	7.34686
0.45	123	98.67	101.374	-2.16841
0.54	77	95.37	101.177	-3.68439
0.69	162	108.21	100.849	6.77454
0.81	395	99.8	100.586	-1.12934
0.82	124	101.47	100.564	0.729464
1.01	112	96.8	100.148	-2.56187

Dose	N	Sample SD	Model Fitted SD
0.37	408	13.3	13.8304
0.4	238	13.31	13.8304
0.45	123	15.7	13.8304
0.54	77	10.31	13.8304
0.69	162	13.72	13.8304
0.81	395	12.2	13.8304
0.82	124	15.5	13.8304
1.01	112	11.16	13.8304

Likelihoods:

Model	Log-Likelihood	# Params	AIC
A1	-6551.38	9	13120.8
A2	-6534.22	16	13100.4
A3	-6551.38	9	13120.8
fitted	-6631.08	3	13268.2
reduced	-6632.06	2	13268.1

Tests of Mean and Variance Fits:

Name	-2 * Log(Likelihood Ratio)	Test d.f.	P-Value
Test 1	195.672	14	0
Test 2	34.3249	7	1.49727e-05
Test 3	34.3249	7	1.49727e-05
Test 4	159.401	6	0

Test 1: Test the null hypothesis that responses and variances don't differ among dose levels (A2 vs R). If this test fails to reject the null hypothesis (p-value > 0.05), there may not be a dose-response.

Test 2: Test the null hypothesis that variances are homogenous (A1 vs A2). If this test fails to reject the null hypothesis (p-value > 0.05), the simpler constant variance model may be appropriate.

Test 3: Test the null hypothesis that the variances are adequately modeled (A3 vs A2). If this test fails to reject the null hypothesis (p-value > 0.05), it may be inferred that the variances have been modeled appropriately.

Test 4: Test the null hypothesis that the model for the mean fits the data (Fitted vs A3). If this test fails to reject the null hypothesis (p-value > 0.1), the user has support for use of the selected model.

Session for MUF and IQ/GCI Excludes ELEMENT

Includes Maternal Urinary Fluoride studies: MIREC, INMA, OCC.

Dataset

Name: MUF and IQ/GCI Excludes ELEMENT

**Dose (mg/L)**	0.37	0.4	0.45	0.69	0.81	0.82
**N**	408	238	123	162	395	124
**Mean ± SD (Scores)**	98.1 ± 13.3	108.07 ± 13.31	98.67 ± 15.7	108.21 ± 13.72	99.8 ± 12.2	101.47 ± 15.5

Test 1 Dose Response: 0
Test 2 Homogeneity of Variance: 0.002
Test 3 Variance Model Selection: 0.002

Settings

Setting	Value
**BMR**	0.5 Standard Deviation
**Distribution**	Normal + Constant variance
**Adverse Direction**	Down (↓)
**Maximum Polynomial Degree**	3
**Confidence Level (one sided)**	0.95

Maximum Likelihood Approach

Model	BMDL	BMD	BMDU	*P*-Value	AIC	Scaled Residual at Control	Scaled Residual near BMD	Recommendation and Notes
Exponential 3	0.852	0.872	0.909	0	11781.206	-6.185	1.14	**Questionable**<br>Residual at control > 2.0<br>Goodness of fit p-value < 0.1<br>Constant variance test failed (Test 2 p-value < 0.05)<br>BMDL/highest dose ratio > 1.0<br>BMD/highest dose ratio > 1.0
Exponential 5	0.868	0.878	0.914	0	11783.205	-6.187	1.129	**Questionable**<br>Residual at control > 2.0<br>Goodness of fit p-value < 0.1<br>Constant variance test failed (Test 2 p-value < 0.05)<br>BMDL/highest dose ratio > 1.0<br>BMD/highest dose ratio > 1.0

Linear	2.071	19.965	-	0	11787.977	-5.016	-0.223	**Questionable**<br>Residual at control > 2.0<br>Goodness of fit p-value < 0.1<br>Constant variance test failed (Test 2 p-value < 0.05)<br>BMDL/highest dose ratio > 1.0<br>BMD/highest dose ratio > 1.0<br>BMD/BMDL ratio > 3.0
Hill	-	-	-	0	11767.579	-1.212	-	**Unusable**<br>Did not successfully execute.
Polynomial 2	0.027	0.034	0.034	0	11745.211	-2.529	-2.529	**Questionable**<br>|Residual near BMD| > 2.0<br>lowest dose/BMDL ratio > 3.0<br>lowest dose/BMDL ratio > 10.0<br>lowest dose/BMD ratio > 3.0<br>lowest dose/BMD ratio > 10.0<br>Residual at control > 2.0<br>Goodness of fit p-value < 0.1<br>Constant variance test failed (Test 2 p-value < 0.05)
Polynomial 3	0.013	0.327	0.333	0	11747.863	-3.176	-3.176	**Questionable**<br>|Residual near BMD| > 2.0<br>lowest dose/BMDL ratio > 3.0<br>lowest dose/BMDL ratio > 10.0<br>Residual at control > 2.0<br>Goodness of fit p-value < 0.1<br>Constant variance test failed (Test 2 p-value < 0.05)<br>BMD/BMDL ratio > 3.0<br>BMD/BMDL ratio > 20.0

Power	0.846	0.871	0.975	0	11781.207	-6.184	1.143	**Questionable**<br>Residual at control > 2.0<br>Goodness of fit p-value < 0.1<br>Constant variance test failed (Test 2 p-value < 0.05)<br>BMDL/highest dose ratio > 1.0<br>BMD/highest dose ratio > 1.0

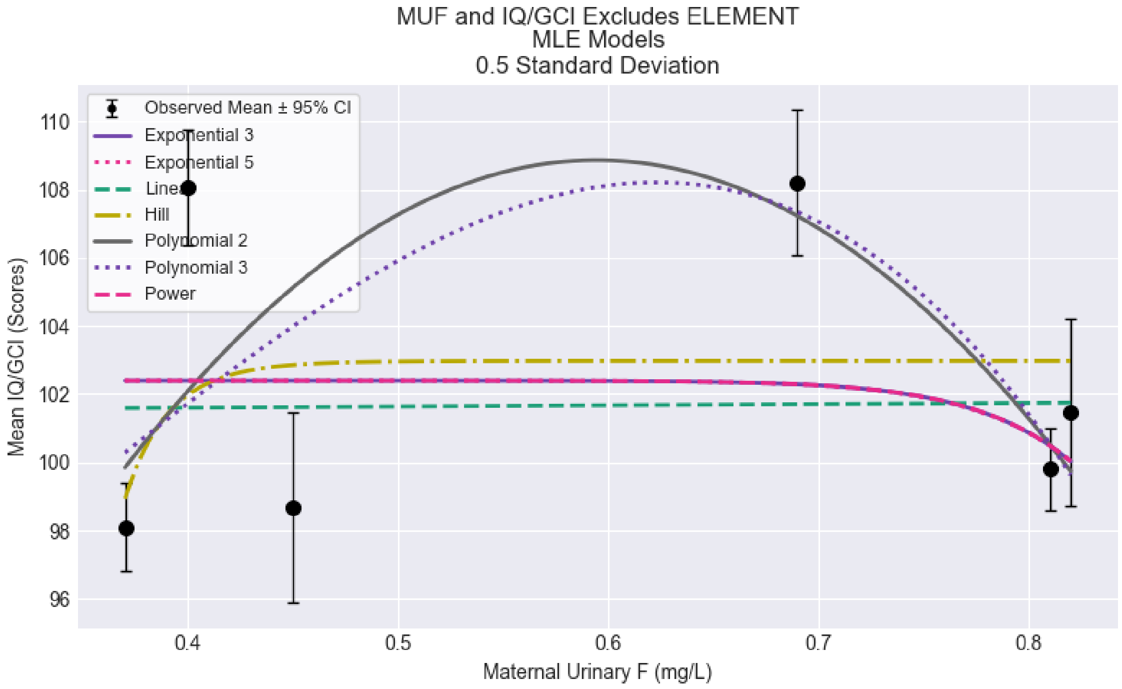

Individual Model Results

Linear Model

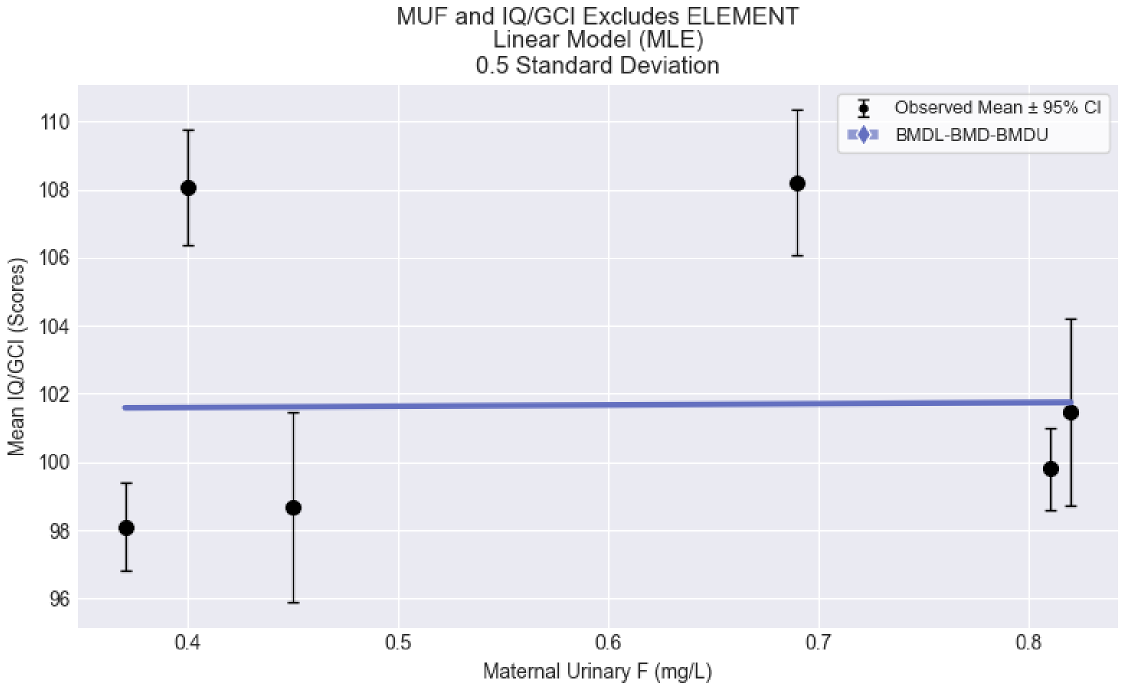


```
          Linear Model
══════════════════════════════════

Version: pybmds 24.1 (bmdscore 24.1)
```

Input Summary:

BMR	0.5 Standard Deviation
Distribution	Normal + Constant variance
Modeling Direction	Down (↓)
Confidence Level (one sided)	0.95
Modeling Approach	MLE
Degree	1

Parameter Settings:

Parameter	Initial	Min	Max
g	0	-1e+06	1e+06
b1	0	-1e+06	1e+06
alpha	0	-18	18

Modeling Summary:

BMD	19.9648
BMDL	2.07053
BMDU	-9999
AIC	11788
Log-Likelihood	-5890.99
P-Value	0
Model d.f.	4

Model Parameters:

Variable	Estimate	On Bound	Std Error
g	101.463	no	0.615308
b1	0.352288	no	0.854655
alpha	197.873	no	1454.13

Goodness of Fit:

Dose	N	Sample Mean	Model Fitted Mean	Scaled Residual
0.37	408	98.1	101.593	-5.01594
0.4	238	108.07	101.604	7.09172
0.45	123	98.67	101.621	-2.32689
0.69	162	108.21	101.706	5.8851
0.81	395	99.8	101.748	-2.75249
0.82	124	101.47	101.752	-0.222969

Dose	N	Sample SD	Model Fitted SD
0.37	408	13.3	14.0667
0.4	238	13.31	14.0667
0.45	123	15.7	14.0667
0.69	162	13.72	14.0667
0.81	395	12.2	14.0667
0.82	124	15.5	14.0667

Likelihoods:

Model	Log-Likelihood	# Params	AIC
A1	-5826.65	7	11667.3
A2	-5817.21	12	11658.4
A3	-5826.65	7	11667.3
fitted	-5890.99	3	11788
reduced	-5891.01	2	11786

Tests of Mean and Variance Fits:

Name	-2 * Log(Likelihood Ratio)	Test d.f.	P-Value
Test 1	147.586	10	0
Test 2	18.8795	5	0.00202408
Test 3	18.8795	5	0.00202408
Test 4	128.671	4	0

Test 1: Test the null hypothesis that responses and variances don't differ among dose levels (A2 vs R). If this test fails to reject the null hypothesis (p-value > 0.05), there may not be a dose-response.

Test 2: Test the null hypothesis that variances are homogenous (A1 vs A2). If this test fails to reject the null hypothesis (p-value > 0.05), the simpler constant variance model may be appropriate.

Test 3: Test the null hypothesis that the variances are adequately modeled (A3 vs A2). If this test fails to reject the null hypothesis (p-value > 0.05), it may be inferred that the variances have been modeled appropriately.

Test 4: Test the null hypothesis that the model for the mean fits the data (Fitted vs A3). If this test fails to reject the null hypothesis (p-value > 0.1), the user has support for use of the selected model.

Supplementary Figure E

Fluoride-IQ BMC

Fluoridation concentration and IQ

Report Generated: 2025-Mar-02 10:03 UTC

BMDS Desktop Version: 24.1 (pybmds 24.1; bmdscore 24.1)

Session for Fluoridation-IQ/GCI Studies

Includes studies conducted in fluoridated communities: Dunedin, MIREC, INMA, APrON, NCOHS.

Dataset

Name: Fluoridation-IQ/GCI Studies

**Dose (mg/L)**	0.1	0.13	0.15	0.16	0.2	0.59	0.7	0.81	0.85	0.86
**N**	123	238	99	68	101	162	295	124	891	194
**Mean ± SD (Scores)**	98.67 ± 15.7	108.07 ± 13.1	98.8 ± 14.5	108.6 ± 12.41	104.62 ± 11.41	108.2 ± 13.7	104.69 ± 14.02	101.47 ± 15.5	100.0 ± 15.1	109.1 ± 12.79

Test 1 Dose Response: 0

Test 2 Homogeneity of Variance: 0.0004

Test 3 Variance Model Selection: 0.0004

Settings

Setting	Value
**BMR**	0.5 Standard Deviation
**Distribution**	Normal + Constant variance
**Adverse Direction**	Down (↓)
**Maximum Polynomial Degree**	3
**Confidence Level (one sided)**	0.95

Maximum Likelihood Approach

Model	BMDL	BMD	BMDU	*P*-Value	AIC	Scaled Residual at Control	Scaled Residual near BMD	Recommendation and Notes
Exponential 3	0.952	0.958	1.094	0	18852.997	-4.789	7.216	**Questionable**<br>|Residual near BMD| > 2.0<br>Residual at control > 2.0<br>Goodness of fit p-value < 0.1<br>Constant variance test failed (Test 2 p-value < 0.05)<br>BMDL/highest dose ratio > 1.0<br>BMD/highest dose ratio > 1.0
Exponential 5	-	-	-	0	18847	-4.893	-	**Unusable**<br>Did not successfully execute.

Linear	1.448	2.159	4.247	0	18863.58	-4.742	6.363	**Questionable**<br>|Residual near BMD| > 2.0<br>Residual at control > 2.0<br>Goodness of fit p-value < 0.1<br>Constant variance test failed (Test 2 p-value < 0.05)<br>BMDL/highest dose ratio > 1.0<br>BMD/highest dose ratio > 1.0
Hill	-	-	-	0	18864.908	-0.059	-	**Unusable**<br>Did not successfully execute.
Polynomial 2	0.202	0.393	0.401	0	18841.321	-3.458	-0.602	**Questionable**<br>Residual at control > 2.0<br>Goodness of fit p-value < 0.1<br>Constant variance test failed (Test 2 p-value < 0.05)
Polynomial 3	0.04	0.155	0.688	0	18840	-3.125	2.007	**Questionable**<br>|Residual near BMD| > 2.0<br>Residual at control > 2.0<br>Goodness of fit p-value < 0.1<br>Constant variance test failed (Test 2 p-value < 0.05)<br>BMD/BMDL ratio > 3.0
Power	0.905	0.956	0.962	0	18853	-4.787	7.214	**Questionable**<br>|Residual near BMD| > 2.0<br>Residual at control > 2.0<br>Goodness of fit p-value < 0.1<br>Constant variance test failed (Test 2 p-value < 0.05)<br>BMDL/highest dose ratio > 1.0<br>BMD/highest dose ratio > 1.0

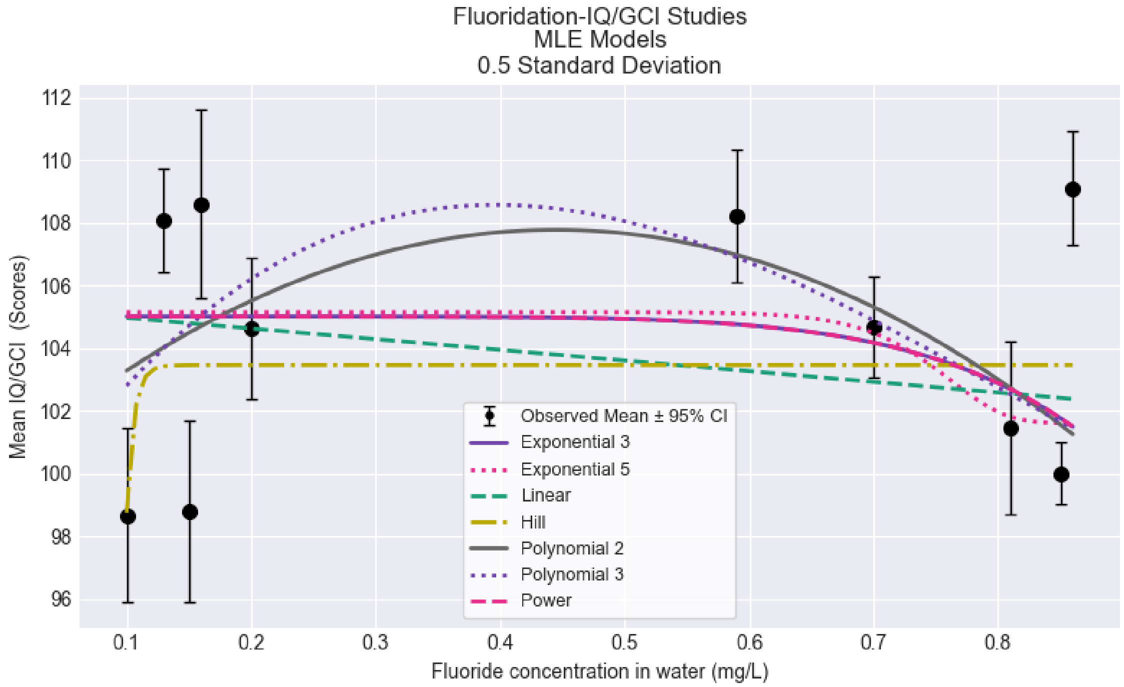

Individual Model Results

Linear Model

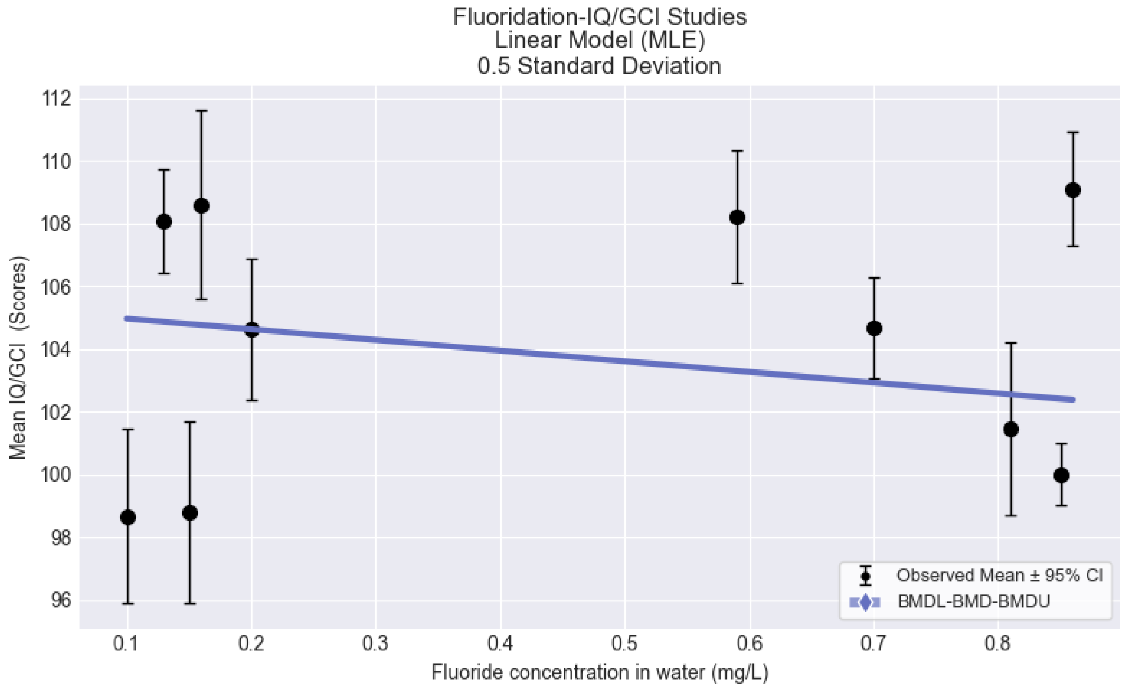


```
        Linear Model
══════════════════════════

Version: pybmds 24.1 (bmdscore 24.1)
```

Input Summary:

BMR	0.5 Standard Deviation
Distribution	Normal + Constant variance
Modeling Direction	Down (↓)
Confidence Level (one sided)	0.95
Modeling Approach	MLE
Degree	1

Parameter Settings:

Parameter	Initial	Min	Max
g	0	-1e+06	1e+06
b1	0	-1e+06	1e+06
alpha	0	-18	18

Modeling Summary:

BMD	2.15909
BMDL	1.44757
BMDU	4.24669
AIC	18863.6
Log-Likelihood	-9428.79
P-Value	0
Model d.f.	8

Model Parameters:

Variable	Estimate	On Bound	Std Error
g	105.306	no	0.699153
b1	-3.40972	no	1.0179
alpha	216.791	no	1387.41

Goodness of Fit:

Dose	N	Sample Mean	Model Fitted Mean	Scaled Residual
0.1	123	98.67	104.965	-4.74159
0.13	238	108.07	104.863	3.36058
0.15	99	98.8	104.794	-4.05086
0.16	68	108.6	104.76	2.15042
0.2	101	104.62	104.624	-0.00271114
0.59	162	108.2	103.294	4.24081
0.7	295	104.69	102.919	2.06577
0.81	124	101.47	102.544	-0.81229
0.85	891	100	102.408	-4.88104
0.86	194	109.1	102.374	6.36306

Dose	N	Sample SD	Model Fitted SD
0.1	123	15.7	14.7238
0.13	238	13.1	14.7238
0.15	99	14.5	14.7238
0.16	68	12.41	14.7238
0.2	101	11.41	14.7238
0.59	162	13.7	14.7238
0.7	295	14.02	14.7238
0.81	124	15.5	14.7238
0.85	891	15.1	14.7238
0.86	194	12.79	14.7238

Likelihoods:

Model	Log-Likelihood	# Params	AIC
A1	-9355.48	11	18733
A2	-9340.39	20	18720.8
A3	-9355.48	11	18733
fitted	-9428.79	3	18863.6
reduced	-9434.39	2	18872.8

Tests of Mean and Variance Fits:

Name	-2 * Log(Likelihood Ratio)	Test d.f.	P-Value
Test 1	188.001	18	0
Test 2	30.1856	9	0.000407925
Test 3	30.1856	9	0.000407925
Test 4	146.62	8	0

Test 1: Test the null hypothesis that responses and variances don't differ among dose levels (A2 vs R). If this test fails to reject the null hypothesis (p-value > 0.05), there may not be a dose-response.

Test 2: Test the null hypothesis that variances are homogenous (A1 vs A2). If this test fails to reject the null hypothesis (p-value > 0.05), the simpler constant variance model may be appropriate.

Test 3: Test the null hypothesis that the variances are adequately modeled (A3 vs A2). If this test fails to reject the null hypothesis (p-value > 0.05), it may be inferred that the variances have been modeled appropriately.

Test 4: Test the null hypothesis that the model for the mean fits the data (Fitted vs A3). If this test fails to reject the null hypothesis (p-value > 0.1), the user has support for use of the selected model.

Citation

U.S. Environmental Protection Agency. (2024). BMDS Desktop (24.1; pybmds 24.1; bmdscore 24.1) [Software].
Available from https://pypi.org/project/bmds-ui/. Accessed March 02, 2025.

Fluoride-IQ BMC – Model for 1 IQ point change is unusable

Fluoridation concentration and IQ

Report Generated: 2025-Feb-25 01:02 UTC

BMDS Desktop Version: 24.1 (pybmds 24.1; bmdscore 24.1)

Session for MUF and IQ/GCI Includes ELEMENT using 1 IQ point change for BMR

Dataset

Name: MUF and IQ/GCI Includes ELEMENT

Dose (mg/L)	N	Mean (Score)	Std. Dev. (Score)
0.37	408	98.1	13.3
0.4	238	108.07	13.31
0.45	123	98.67	15.7
0.54	77	95.37	10.31
0.69	162	108.21	13.72
0.81	395	99.8	12.2
0.82	124	101.47	15.5
1.01	112	96.8	11.16

Settings

Setting	Value
**BMR**	1.0 Point Estimation
**Distribution**	Normal + Nonconstant variance
**Adverse Direction**	Up (↑)
**Maximum Polynomial Degree**	3
**Confidence Level (one sided)**	0.95

Maximum Likelihood Approach

Model	BMDL	BMD	BMDU	*P*-Value	AIC	Scaled Residual at Control	Scaled Residual near BMD	Recommendation and Notes
Exponential 3	-	-	-	-1	-	0	-	**Unusable** Did not successfully execute.
Exponential 5	-	-	-	0	13248.34	0.005	-	**Unusable** Did not successfully execute.
Hill	-	0.013	-	0	13276	-4.277	-4.277	**Unusable** Did not successfully execute.
Polynomial 2	-	-	-	0	13270	-4.287	-	**Unusable** Did not successfully execute.
Polynomial 3	-	-	-	0	13268	-4.287	-	**Unusable** Did not successfully execute.
Power	-	1.391	-	0	13260.624	-5.138	0.198	**Unusable** Did not successfully execute.
Linear	-	-	-	0	13266.254	-5.42	-	**Unusable** Did not successfully execute.

Source: EPA Document# 740-P1-8001

United States Office of Chemical Safety and Environmental Protection Agency Pollution Prevention, Page 231

H.5 Data Quality Criteria

Table H-8. Serious Flaws that Would Make Epidemiological Studies Unacceptable for Use in the Hazard Assessment

Optimization of the list of serious flaws may occur after pilot calibration exercises.

Domain	Metric	Description of Serious Flaw(s) in Data Source
Study Participation	Participant Selection	For all study types: The reported information indicates that selection in or out of the study (or analysis sample) and participation was likely to be significantly biased (i.e., the exposure-outcome distribution of the participants is likely not representative of the exposure-outcome distributions in the overall population of eligible persons.)
	Attrition	For cohort studies: The loss of subjects (i.e., incomplete outcome data) was large and unacceptably handled (as described above in the low confidence category) (Source: OHAT).<br>OR<br>Numbers of individuals were not reported at important stages of study (e.g., numbers of eligible participants included in the study or analysis sample, completing follow-up, and analyzed). Reasons were not provided for non-participation at each stage [STROBE Checklist Item 13 (Von Elm et al., 2008)].
		For case-control and cross-sectional studies: The exclusion of subjects from analyses was large and unacceptably handled (as described above in the low confidence category).<br>OR<br>Reasons were not provided for non-participation at each stage [STROBE Checklist Item 13 (Von Elm et al., 2008)].
	Comparison Group	For cohort studies: Subjects in all exposure groups were not similar, recruited within very different time frames, or had the very different participation/ response rates (NTP, 2015a).<br>OR<br>Information was not reported to determine if participants in all exposure groups were similar [STROBE Checklist 6 (Von Elm et al., 2008)]
		For case-control studies: Controls were drawn from a very dissimilar population than cases or recruited within very different time frames (NTP, 2015a).<br>OR<br>Rationale and/or methods for case and control selection, matching criteria including number of controls per case (if relevant) were not reported [STROBE Checklist 6 (Von Elm et al., 2008)].

		For cross-sectional studies: Subjects in all exposure groups were not similar, recruited within very different time frames, or had the very different participation/response rates (NTP, 2015a).

Domain	Metric	Description of Serious Flaw(s) in Data Source
		OR<br>Sources and methods of selection of participants in all exposure groups were not reported [STROBE Checklist 6 (Von Elm et al., 2008)].
Exposure Characterization	Measurement of Exposure	For all study types: Exposure variables were not well defined, and sources of data and detailed methods of exposure assessment were not reported [STROBE Checklist 7 and 8 (Von Elm et al., 2008)].<br>OR<br>Exposure was assessed using methods known or suspected to have poor validity (Source: OHAT).<br>OR<br>There is evidence of substantial exposure misclassification that would significantly alter results.
	Exposure Levels	For all study types: The levels of exposure are not sufficient or adequate (as defined above) to detect an effect of exposure (Cooper et al., 2016).<br>OR<br>No description is provided on the levels or range of exposure.
	Temporality	For all study types: Study lacks an established time order, such that exposure is not likely to have occurred prior to outcome (Lakind et al., 2014).<br>OR<br>Exposures clearly fell outside of relevant exposure window for the outcome of interest.<br>OR<br>For each variable of interest (outcome and predictor), sources of data and details of methods of assessment were not reported (e.g., periods of exposure, dates of outcome ascertainment, etc.) [STROBE Checklist 8 (Von Elm et al., 2008)].
Outcome Assessment	Outcome measurement or characterization	For all study types: Numbers of outcome events or summary measures, or diagnostic criteria were not defined or reported [STROBE Checklist 15 (Von Elm et al., 2008)].
Potential		For cohort and cross-sectional studies: The distribution of primary covariates (excluding co-exposures) and known confounders differed significantly between the exposure groups<br>OR<br>Confounding was demonstrated and was not appropriately adjusted for in the final analyses (NTP, 2015a).

Confounding/Variable Control	Covariate adjustment	For case-control studies: The distribution of primary covariates (excluding co-exposures) and known confounders differed significantly between cases and controls.<br>OR<br>Confounding was demonstrated and was not appropriately adjusted for in the final analyses (NTP, 2015a).

Domain	Metric	Description of Serious Flaw(s) in Data Source
	Covariate characterization	For all study types: Primary covariates (excluding co-exposures) and confounders were not assessed.
	Co-exposure Confounding/ Moderation/ Mediation	For cohort and cross-sectional studies: There is direct evidence that there was an unbalanced provision of additional co-exposures across the primary study groups, which were not appropriately adjusted for.
		For case-control studies: There is direct evidence that there was an unbalanced provision of additional co-exposures across cases and controls, which were not appropriately adjusted for, and significant indication a biased exposure-outcome association.
Analysis	Study design and methods	For all study types: The study design chosen was not appropriate for the research question.<br>OR<br>Inappropriate statistical analyses were applied to assess the research questions.
	Statistical power (sensitivity)	For cohort and cross-sectional studies: The number of participants are inadequate to detect an effect in the exposed population and/or subgroups of the total population.
		For case-control studies: The number of cases and controls are inadequate to detect an effect in the exposed population and/or subgroups of the total population.
Other (if applicable) Considerations for Biomarker Selection and Measurement (Lakind et al., 2014)	Use of Biomarker of Exposure	Biomarker in a specified matrix is a poor surrogate (low accuracy and precision) for exposure/dose.
	Effect biomarker	Biomarker has undetermined consequences (e.g., biomarker is not specific to a health outcome).
	Method sensitivity	Frequency of detection too low to address the research hypothesis.<br>OR<br>LOD/LOQ (value or %) are not stated.
	Biomarker stability	Samples with either unknown storage history and/or no stability data for target analytes and high likelihood of instability for the biomarker under consideration.
	Sample contamination	There are known contamination issues and no documentation that the issues were addressed.
	Method requirements	Instrumentation that only allows for possible quantification of the biomarker, but the method has known interferants (e.g., GC–FID, spectroscopy).

	Matrix adjustment	If applicable for the biomarker under consideration, no established method for matrix adjustment was conducted.

H.4.2 Calculation of Overall Study Score - Page 227

EPA/OPPT plans to use data with an overall quality level of *High, Medium*, or *Low* confidence to quantitatively or qualitatively support the risk evaluations, but does not plan to use data rated as *Unacceptable*. Studies with any single metric scored as 4 will be automatically assigned an overall quality score of *Unacceptable* and further evaluation of the remaining metrics is not necessary. An *Unacceptable* score means that serious flaws are noted in the domain metric that consequently make the data unusable (or invalid).

Table H-4. Epidemiology Metrics with Greater Importance in the Evaluation and Rationale for Selection

**Domain**	**Critical Metrics with Higher Weighting Factors (Metric Number)** [a]	**Rationale**
Exposure characterization	Measurement of Exposure (Metric 4)	**The exposure of interest of should be well-defined and measured in a manner that is accurate, precise, and reliable to ensure the internal and external validity of the study findings (Blumenthal et al. 2001, Nieuwenhuijsen 2015).**

Assessment of Spot maternal urinary fluoride: 1-3 spot maternal urinary fluoride sample as a proxy for long-term fetal fluoride exposure is not accurate, precise, and reliable measure to ensure internal and external validity of the study findings.

SUPPLEMENTARY TABLE S1. Data Quality Assessment

Table S1. Data quality assessment. Serious Flaws that Would Make Epidemiological Studies Unacceptable for Use in the Hazard Assessment				
**Domain and Metric**	**ELEMENT**	**MIREC**	**INMA**	**OCC**
**1. Study participation**<br><br>Participant Selection<br><br>The reported information indicates that selection in or out of the study (or analysis sample) and participation was likely to be significantly biased.	Secondary data analysis project.<br><br>Multiple groups recruited from three hospitals during different time periods that included two arms of a clinical trial.<br><br>1. Cohort 1 and Cohort 2B recruited participants at birth and did not have archived maternal-pregnancy urine samples required for this analysis; they were thus excluded.<br><br>2.Mothers for Cohort 2A (n = 327) and 3 (n = 670) were all recruited from the same three hospitals in Mexico city that serve low-to-moderate income populations.<br><br>3.Cohort 3 mothers were pregnant women (≤14 wk of gestation) recruited from 2001 to 2003 for a randomized trial of the effect of calcium supplementation during pregnancy on maternal blood lead levels.<br>Mothers with fluoride and creatinine data =512<br><br>Children with data on complete GCI and covariates = 287<br>Children with data on complete IQ and covariates = 211	Secondary data analysis project.<br><br>Between 2008 and 2011, the Maternal-Infant Research on Environmental Chemicals (MIREC) program recruited 2001 pregnant women from 10 cities across Canada.<br><br>A subset of 610 children in the MIREC Study was evaluated for the developmental phase of the study at ages 3 to 4 years; these children were recruited from 6 of 10 cities included in the original cohort: Vancouver, Montreal, Kingston, Toronto, Hamilton, and Halifax.	Secondary data analysis project.<br><br>All pregnant women were contacted. Recruitment took place during the first pre-natal visit (10-13 weeks of gestation) in the main public hospital or health center in Gipuzkoa. 68% participated.<br><br>In total, 638 pregnant women met the inclusion criteria and agreed to be enrolled in the INMA study. Of the 612 children born, 483 (78.9%) underwent neuropsychological testing at the age of 1 and 379 (61.9%) at the age of 4.	Secondary data analysis project<br><br>All new pregnant women residing in Odense municipality were contacted between 2010 and 2012; 2874 of the 4017 women agreed to be enrolled in the OCC, while 374 dropped out before and after giving birth.12 The present study population included 837 singleton mother–child pairs with results on child IQ, a maternal urine sample analyzed for fluoride, and information about parental education, child sex and preterm birth.
Attrition<br>For cohort studies: The loss of subjects (i.e., incomplete outcome data) was large and unacceptably handled (as described above in the low confidence category) (Source: OHAT).	Cohort 2A - 131 at age 4; 93 at 6-12<br>Cohort 3 Ca - 134 at age 4; 104 at 6-12<br>Cohort 3 P - 121 at age 4; 81 at 6-12	Only one assessment	Only one assessment	Only one assessment
Comparison group<br>For cohort studies: Subjects in all exposure groups were not similar, recruited within very different time	Recruited within very different times	Comparability of six groups not presented	One geographic region	One geographic region

SUPPLEMENTARY TABLE S1. Data Quality Assessment

frames, or had the very different participation/ response rates (NTP, 2015a).				
**Confidence**	**Unacceptable**	**Unacceptable**	**Medium**	**Low**
**2. Exposure characterization**<br><br>Measurement of Exposure<br><br>Exposure was assessed using methods known or suspected to have poor validity (Source: OHAT).<br>OR<br>There is evidence of substantial exposure misclassification that would significantly alter results.<br><br>Biomarker in a specified matrix is a poor surrogate (low accuracy and precision) for exposure/dose.<br><br>Exposure Levels<br>For all study types: The levels of exposure are not sufficient or adequate (as defined above) to detect an effect of exposure (Cooper et al., 2016). OR<br>No description is provided on the levels or range of exposure.<br><br>Temporality	Spot urinary F is not a valid method at the individual level or fetal F exposure<br><br>1-3 samples of spot urinary F validation showed there was no association between plasma F and MUF.<br><br>The correlation between trimesters is weak.<br><br>Postnatal exposure was not included in the Goodman et al. analysis. Bashash et al. showed it attenuated the effect and the results were not statistically significant.<br><br>D. Thomas showed that the effect was positive.<br><br>Whether IQ deficits occurred during high exposure is not known.<br>Cumulative exposure was not documented	Spot urinary F is not a valid method at the individual level or fetal F exposure<br><br>3 trimester specific samples data were averaged.<br>The authors did not demonstrate that three samples are sufficient to demonstrate validity.<br><br>Green et al. did not include postnatal exposure.<br><br>Till et al. and Farmus et al. showed that the addition of postnatal exposure altered the results dramatically.<br><br>Whether IQ deficits occurred during high exposure is not known<br>Cumulative exposure was not documented	Spot urinary F is not a valid method at the individual level or fetal F exposure<br><br>First and third trimester data collected<br><br>The authors did not demonstrate that two samples are sufficient to demonstrate validity.<br><br>Postnatal exposure data not available.<br><br>Whether IQ deficits occurred during high exposure is not known<br>Cumulative exposure was not documented	A single 24 hr. and spot urinary F is not a valid method at the individual level or fetal F exposure<br><br>The authors did not demonstrate that the samples are sufficient to demonstrate validity.<br><br>Postnatal exposure data not available.<br><br>Whether IQ deficits occurred during high exposure is not known<br>Cumulative exposure was not documented
**Confidence**	**Unacceptable**	**Unacceptable**	**Unacceptable**	**Unacceptable**
**Measurement of exposure using fluoride concentration or maternal spot urinary F** to classify groups as high or low exposure for SMD analysis and dose response	Spot urinary F was used at the group level	Records of fluoride in drinking water were matched to individual home addresses.	Records of fluoride in drinking water at the group level	The authors did not provide the data. Spot maternal urinary fluoride at the group level was used on abstracted data.
**Confidence**	**Medium**	**High**	**Medium**	**Medium**
**3. Outcome measurement or characterization**<br>For all study types: Numbers of outcome events or summary measures, or diagnostic criteria were	Child neurobehavioral outcomes were measured at ages 1, 2 and 3 using MDI but not published. This analysis suggested that maternal intake of fluoride during pregnancy did not have	The IQ test methods is not discussed.<br><br>Six research assistants, each assigned to a city, conducted the assessments. Inter-examiner reliability assessment	"All testing was carried out in local health centers in the presence of the mother, father or another caregiver by specially trained neuropsychologists who were blinded to the child's F	Details not reported.<br><br>Regression coefficient was adjusted for examiner.

SUPPLEMENTARY TABLE S1. Data Quality Assessment

not defined or reported [STROBE Checklist 15 (Von Elm et al., 2008)].	an effect.<br>An analysis of data on 6-15 year old children (N= 550) is not published.<br><br>“Both tests were administered by a team of three psychologists who were trained and supervised by an experienced developmental psychol-ogist (L.S.). This team of three psychologists applied all of the McCarthy tests as well as the WASI-FSIQ tests. At the time of follow-up visits (age 4 and 6–12 y), each child was evaluated by one of the psychologists who was blind to the children’s fluoride exposure.”<br><br>The mean observer–examiner correlation was 0.99 Inter-examiner reliability was not examined on the WASI test. All raw scores were standardized for age and sex.	was not reported.	exposure status. To limit inter-observer variability, we applied a strict protocol, including training sessions in which inter-observer differences were discussed (Guxens et al., 2012). The scores according to the age were standardized to a mean of 100 points with a standard deviation of 15 points.”	
**Confidence**	**Unacceptable**	**Unacceptable**	**Medium**	**Medium**
**4. Potential Confounding/Variable Control**	Adjusted for gestational age, weight at birth, sex, parity (being the first child),age at outcome measurement, and maternal characteristics including smoking history (ever smoked vs.nonsmoker), marital status (married vs. others), age at delivery, IQ, education, and cohort(Cohort3-Ca, Cohort3- placebo and Cohort2A).<br><br>Extensive sensitivity analysis.<br>**However, not adjusted for salt intake, a potential confounder.**<br><br>Prediction model	Adjusted for city, HOME score, maternal education, race/ethnicity, and including child sex interaction.<br><br>The number of smokers was small. However, it was added to some models.<br><br>The following variables were available but not included - BMI, paternal variables, maternal chronic condition during pregnancy and birth country; breastfeeding duration; and time of void and time since last void.<br><br>**Not adjusted for examiner.**<br><br>Prediction model	Adjusted by age of the child at the time of the test (only for McCarthy), order of the child (between siblings), nursery at 14 months, breastfeeding, maternal social class, maternal IQ and smoking.<br>Extensive additional analyses included neurotoxic substances detected in maternal urine samples during pregnancy, such as total As (µg/g) and manganese (Mn, µg/g), both adjusted for creatinine, and Hg in umbilical cord blood (µg/l), cord blood Pb and Iodine.<br><br>Causal model	“The simple model is adjusted for parental education and preterm birth. The comprehensive model accounts also for age at the time of testing, examiner, breastfeeding duration, school grade, school type and smoking and alcohol habits of the mother during pregnancy.”<br><br>Prediction model
**Confidence**	**Low**	**Low**	**High**	**Low**
**5. Analysis**	Cross sectional analysis of a cohort<br><br>Our overall strategy for selecting covariates for adjustment was to identify those that are well	Cross sectional analysis of a cohort<br><br>We selected covariates from a set of established factors associated with fluoride metabolism (eg,	Cross sectional analysis of a cohort<br><br>Adjusted by age of the child at the time of the test (only for McCarthy), order of the child (between siblings),	Cross sectional analysis of a cohort<br><br>The description is sketchy. The authors did not follow the STROBE checklist and present unadjusted model

SUPPLEMENTARY TABLE S1. Data Quality Assessment

	known to have potential associ- ations with either fluoride exposure or cognitive outcomes and/or are typically adjusted for as potential confounders in analyses of environmental toxicants and cognition. All models were adjusted for gestational age at birth (in weeks), birthweight (kilograms), birth order (first born yes vs. no), sex, and child's age at the time of the neurocognitive test (in years). All models were also adjusted for maternal characteristics including marital status (married vs. others), smoking history (ever-smoker vs. never- smoker), age at delivery, IQ, and education (itself also a proxy for socioeconomic status). Finally, all models adjusted for potential cohort effects by including indicator variables denoting from which cohort (Cohort 2A, Cohort 3 + Ca supplement, and Cohort 3 -placebo) the participants came.	time of void and time since last void) and children's intellectual abilities (eg, child sex, maternal age, gestational age, and parity) (Table 1). Mother's race/ ethnicity was coded as white or other, and maternal educa- tion was coded as either bachelor's degree or higher or trade school diploma or lower. The quality of a child's home environment was measured by the Home Observation for Measurement of the Environment (HOME)– Revised Edition19 on a continuous scale. We also controlled for city and, in some models, included self-reported exposure to secondhand smoke (yes/no) as a covariate.	nursery at 14 months, breastfeeding, maternal social class, IQ and smoking.<br><br>Additional analyses including other variables like other neuro- toxicants (As, Mn, Pb, Hg and As x Pb), iodine, quality child's family context (HES) and deprivation index instead of maternal social class, were carried out.	The simple model is adjusted for parental education and preterm birth. The comprehensive model accounts also for age at the time of testing, examiner, breastfeeding duration, school grade, school type and smoking and alcohol habits of the mother during pregnancy.
Analysis – Consideration of design effect resulting from cluster sampling	Not considered	Not considered<br>NASEM:<br>Green et al. (2019) accounted for community-level effects by adjusting for city in their analysis, but it was unclear how this was done. If they treated city as a random effect, their analytic methods were appropriate. However, if they treated city as a fixed effect, their exposure-effect estimates might be biased. When exposure levels are determined at the group (such as city) level, fixedeffect models do not properly separate exposure effects from group effects, and this results in biased estimates and inflated type I errors (Zucker 1990). Although Green et al. (2019) used individual- level exposure rather than city-level exposure, the fixed-effect model could still produce biased estimates if the exposure levels within a city are highly correlated; this might be expected given that some cities.	Not applicable	Not applicable

SUPPLEMENTARY TABLE S1. Data Quality Assessment

		were fully on fluoridated water and others were not.		
**Confidence**	Low	**Unacceptable**	**Medium**	**Medium**
**6. Other (if applicable) Considerations for Biomarker Selection and Measurement (Lakind et al., 2014)** Use of Biomarker of ExposureBiomarker in a specified matrix is a poor surrogate (low accuracy and precision) for exposure/dose.	Maternal spot urinary F biomarker is a poor surrogate (low accuracy and precision) for long-term fetal F exposure/dose.<br><br>“Fluoride is not homeostatically controlled [13]. This means that fluoride levels in body fluids or tissues will show a range of values that will be highly variable and dependent on short- and long- term fluoride exposure. Thus, a ‘normal’ (as used in clinical chemistry) urinary fluoride excretion range of values cannot be established.”<br><br>“While fluoride concentrations in plasma, saliva and urine have some ability to predict fluoride exposure, data are, at present, **insufficient to recommend fluoride concentration in these body fluids as viable biomarkers of contemporary fluoride exposure for individuals.** Fluoride concentration in urine can be considered a **useful biomarker of contemporary fluoride exposure for groups of people**, and normal values have been published.” Rugg- Gunn et al. Contemporary Biological Markers of Exposure to Fluoride. Fluoride Intake, Metabolism and Toxicity. Buzalaf MAR (ed): Fluoride and the Oral Environment. Monogr Oral Sci. Basel, Karger, 2011, vol 22, pp 37–51.<br><br>“Because of substantial within- and between-individual variation in urinary flow and creatinine excretion rates, as well as the rapid urinary elimination pharmacokinetics of fluoride, concentrations of fluoride in individual spot samples may vary substantially even when underlying exposures rates are consistent<br>and within the exposure guidance values. **For this reason, we recommend that the BE values derived here be applied to the evaluation of central tendency estimates for populations, rather than to the evaluation of individual spot sample concentrations**.” Aylward et al. http://dx.doi.org/10.1016/j.yrtph.2015.04.005<br><br>“Several areas of uncertainty are associated with the use of urinary fluoride as a biomarker of fluoride exposure. These uncertainties, listed below, should be<br>considered when planning, conducting and interpreting the findings of studies of urinary fluoride excretion.” W.H.O. Basic methods for assessment of renal fluoride excretion in community prevention programmes for oral health, 2014			
**Overall Confidence**	Unacceptable	Unacceptable	Unacceptable	Unacceptable
**Note: We have copied some text directly from the papers to provide an accurate description of the study.**				

Unpublished findings from the ELEMENT Study: There may be systematic differences between reported and unreported results.
D. Thomas analyzed two cohorts of the ELEMENT project for her thesis. “Participants for the prenatal exposure portion of this project were recruited between 1997 and 2005. Each woman contributed between one and three urine samples and between one and three plasma samples during the course of her pregnancy. After the delivery, the infants were administered neurobehavioral tests at ages one, two and three to continually assess their development (Mental Development Index). Participants for the concurrent fluoride and neurobehavior portion of this project were a subset of children recruited from ELEMENT for a follow-up study on lead and neurobehavior. 1272 of the mother-infant pairs eligible to participate in the follow-up were approached between 2008 and 2010, urine samples were available for 653 pairs.”

SUPPLEMENTARY TABLE S1. Data Quality Assessment

"We measured urinary fluoride in 695 women, and of these women, 431 women had a child with at least one MDI score: 355 children had an MDI score at age 1, 397 at age 2, and 358 at age 3. Plasma fluoride was measured in 352 women, and 194 of these women had a child with atleast one MDI score: 194 children had an MDI score at age 1, 184 children at age 2 and 167 children at age 3. Neither maternal urinary or plasma fluoride was associated with offspring MDI scores (βurine = -0.631, p=0.391; βplasma= -0.0031, p=0.650.) 38 The effect of urinary or plasma fluoride on MDI also did not significantly change between the ages of 1, 2 and 3."

"Fluoride measurements were drawn from 6-15 year old children (N= 550) during their baseline visit in the Cholesterol study… To assess the adjusted effect of child's fluoride levels on cognitive function, we ran GAM models, first in the overall population and then stratified by gender. In the overall population, urinary fluoride appears to have no significant impact on total WASI scores (β =1.32, p=0.33), but this association changes once the models are separated by male and female children. Male children showed a significantly positive trend (β=3.81, p=0.05), and females showing a negative trend that was not significant (β= -1.57, p=0.39)."

Inconsistent findings between Green et al. and Farmus et al. (MIREC):

Green et al. 2019	There was no association between maternal urinary F and IQ. However, when stratified by sex, an increase in maternal urinary F was associated with a decrease in IQ in boys. In girls, there was an increase in IQ, but it was not statistically significant. The authors reported a sex-specific interaction effect.
Farmus et al. 2021	"The association between MUF and IQ scores did not differ significantly across trimesters."<br>There was no sex-specific effect associated with MUF and FSIQ in any of the trimester windows.<br><br>'The accepted (pre-proof) version, made available online 27 May 2021, included the following sentence that referred to a supplemental analysis: *"However, exposures do not significantly associate with IQ outcomes once city is controlled and FDR is applied."*<br><br>Addendum:<br>**Critical Windows of Fluoride Neurotoxicity in Canadian Children**<br>doi: 10.1016/j.envres.2021.111315

SUPPLEMENTARY TABLE S1. Data Quality Assessment

Domain and Metric	Broadbent et al.	Dewey et al.	Do et al.
**1. Study participation**<br><br>Participant Selection<br><br>The reported information indicates that selection in or out of the study (or analysis sample) and participation was likely to be significantly biased.	Secondary data analysis project of a birth cohort<br><br>“Participants were members of the Dunedin Multidisciplinary Health and Development Study, a longitudinal investigation of the health and behavior of a complete birth cohort of consecutive births between April 1, 1972, and March 31, 1973, in Dunedin, New Zealand. The cohort of 1037 children (91% of eligible births; 52% boys) was constituted at age 3 years. Cohort families represent the full range of socioeconomic status (SES) in the general population of New Zealand’s South Island and are primarily of white European ancestry. We conducted follow-up assessments with informed consent at 5, 7, 9, 11, 13, 15, 18, 21, 26, 32, and most recently at 38 years of age, when 95.4% of the 1007 living study members underwent assessment in 2010 to 2012.	Secondary data analysis project of birth cohorts<br><br>“Between 2009 and 2012, the APrON study recruited 1969 pregnant women from Calgary, Alberta, Canada. Women were eligible if they could communicate in English, were <27 weeks gestational age and were ≥16 years of age. A subset of 616 maternal-child pairs from Calgary whose children participated in cognitive and executive function assessments at 3 to 5 years of age (M=4.24 SD=0.51) participated in this study.”	Based on a large existing population-based study, the Australia’s National Child Oral Health Study (NCOHS) 2012–2014<br><br>“ 2020–2021, a first follow-up of the NCOHS sample investigated associations between exposure to fluoridation and child behavioral development (Do et al. 2023). As part of this follow-up study, in 2022–2023 a subset of NCOHS participants who had turned 16+ y was recruited and completed the WAIS-IV. Those who were identified as having dental fluorosis in theNCOHS clinical assessment were first invited to participate in the intelligence assessment. A random sample of participants without dental fluorosis with similar age (±1 y) and same sex were subsequently selected and invited, with a ratio of 4 to 1 (the prevalence of dental fluorosis was 18%).”<br><br>“We used SAS Proc POWER to estimate a required sample to test a hypothesis of noninferiority in FSIQ scores”
Attrition<br>For cohort studies: The loss of subjects (i.e., incomplete outcome data) was large and unacceptably handled (as described above in the low confidence category) (Source: OHAT).	Only one assessment<br><br>Data on IQ were available for 992 and 942 study members in childhood and adulthood, respectively.	Only one assessment<br><br>The authors did not discuss if the subset was similar to those recruited for the study.<br><br>“Few differences in sample characteristics were found between the overall sample and the fluoride exposure groups.”	Only one assessment
Comparison group<br>For cohort studies: Subjects in all exposure groups were not similar, recruited within very different time frames, or had the very different participation/ response rates (NTP, 2015a).	All participants were recruited from the same region	The not exposed group had a lower prevalence of women born in Canada (71.29 %) compared to the partially (83.64%) and fully exposed (84.75 %) groups, and a lower proportion of women who reported smoking prior to pregnancy (13.86 %) compared to the partially (25.0 %) and fully exposed (27.12 %) groups.	There were some differences between groups regarding education and income.
**Confidence**	Medium	Medium	Medium
**2. Exposure characterization**<br><br>Measurement of Exposure	Ecologic exposure measure<br><br>Most suburbs of Dunedin have had CWF since 1967, but certain suburbs	Ecologic exposure measure<br><br>Water fluoride records are available.	Individual level data used<br><br>The NCOHS questionnaire collected a detailed residential history from birth to the time of the survey and included consumption

SUPPLEMENTARY TABLE S1. Data Quality Assessment

Exposure was assessed using methods known or suspected to have poor validity (Source: OHAT).<br>OR<br>There is evidence of substantial exposure misclassification that would significantly alter results.<br><br>Biomarker in a specified matrix is a poor surrogate (low accuracy and precision) for exposure/dose.<br><br><u>Exposure Levels</u><br>For all study types: The levels of exposure are not sufficient or adequate (as defined above) to detect an effect of exposure (Cooper et al., 2016). OR<br>No description is provided on the levels or range of exposure.<br><br>Temporality	remain unfluoridated. We report residence in an area with or without CWF (0.7---1.0 ppm and 0.0---0.3 ppm fluoride, respectively) coded from residential address data to age 5 years (n = 922), or to age 3 years (n = 103) where residence data from age 5 years were unavailable (area of residence for 2 study members could not be coded at either age).<br><br>Difficult to determine	Difficult to determine	of public water and other water sources. The residential history of participants has been linked to the postcode-level fluoride concentration in public water database that was updated yearly by water authorities.
**Confidence**	Low	Low	Medium
**<u>3.</u> Outcome measurement or characterization**<br>For all study types: Numbers of outcome events or summary measures, or diagnostic criteria were not defined or reported [STROBE Checklist 15 (Von Elm et al., 2008)].	We assessed childhood IQ for each study member at ages 7, 9, 11, and 13 years by means of the Wechsler Adult Intelligence Scale-Revised (WISC-R).23 The IQs determined at these 4 ages were averaged into 1 measure and standardized.<br><br>Tests were administered in the morning by trained psychometrists who were blind to the study members' previous IQ data. In addition, examiners were unaware of the CWF status of participants' area of residence.	Children's Full Scale IQ (FSIQ) on the Wechsler Preschool and Primary Scale of Intelligence Fourth Edition: Canadian (WPPSI-IVCND) was our primary outcome.<br><br>The IQ assessment methods and the number of assessors are not discussed.	Qualified psychologists, employed to administer the WAIS-IV, underwent training and administration compliance testing ran by J.K.K., a neuropsychologist with extensive experience in Wechsler intelligence scales. All participants were administered the WAIS-IV test one-on-one with a trained psychologist<br>in standardized conditions for the psychological assessment (in a quiet room, with participants facing a monotonic wall without any distractions). The psychologists and participants were not aware of the exposure status of the participants.
**Confidence**	High	Low	High
**4. Potential Confounding/Variable Control**	Variables considered as prior causes common to both low IQ and adult mental disorders were included as confounders in our models, as done in previous research.25 Childhood measures included SES, birth weight, and breastfeeding. SES was based on parental occupation (and the	Potential covariates were selected from established factors associated with children's intellectual abilities and executive functioning (i.e., maternal age, sociodemographic factors, maternal pre-pregnancy<br>BMI, maternal pre-pregnancy smoking, maternal depression, child sex, and infant gestational age at birth). As performance on the Gift Delay,	Covariates used in the multivariable regression models were selected as potentially influencing the associations between fluoride exposures and the outcomes (see the directed acyclic graph in Appendix Fig. 2). Covariates were age at follow-up, sex, household income, parental education and country of birth, participants' neurodevelopmental diagnosis (attentiondeficit hyperactivity disorder, autism spectrum disorder, dyslexia, dyscalculia), breastfeeding duration (never, up to 6 mo, 6

SUPPLEMENTARY TABLE S1. Data Quality Assessment

	educational level and income associated with that occupation in the New Zealand census)26 and categorized into 3 groups. Low birth weight was defined as birth weight below 2.50 kilograms. Breastfeeding was defined as breastfeeding for 4 weeks or more.	DCCS, and Boy-Girl Stroop were not age standardized, child age was included as a covariate. The covariates were coded as follows: maternal education (i.e., bachelor's degree or higher, trade school diploma or lower), maternal marital status (i.e., married/living with a partner, single/separated/ divorced/widowed), annual household income (i.e., <$70,000, >$70,000),maternal birthplace (i.e., born in Canada or not), maternal parity	to 24 mo, and 24+ mo), and toothbrushing with fluoride toothpaste collected retrospectively for age 2 y (<2 times/day, 2+ times/day).
**Confidence**	High	High	High
**5. Analysis**	Appropriate statistical analyses were applied to assess the research questions. Additional analyses were published as part of a letter to the editor to address concerns.	Appropriate statistical analyses were applied to assess the research questions.	Appropriate statistical analyses were applied to assess the research questions.
Analysis – Consideration of design effect resulting from cluster sampling			
**Confidence**	High	High	High
**6. Other (if applicable) Considerations for Biomarker Selection and Measurement (Lakind et al., 2014)** Use of Biomarker of ExposureBiomarker in a specified matrix is a poor surrogate (low accuracy and precision) for exposure/dose.	Not applicable	Not applicable	
**Overall Confidence**	**Acceptable**	**Acceptable**	**Acceptable**

Supplementary Figure F.

Fluoride concentration and Response in MUF Cohort Studies (Mean IQ /GCI) and Dean's Severe Dental Fluorosis (%)

Severe dental fluorosis (Dean's Study)

The model fits the data

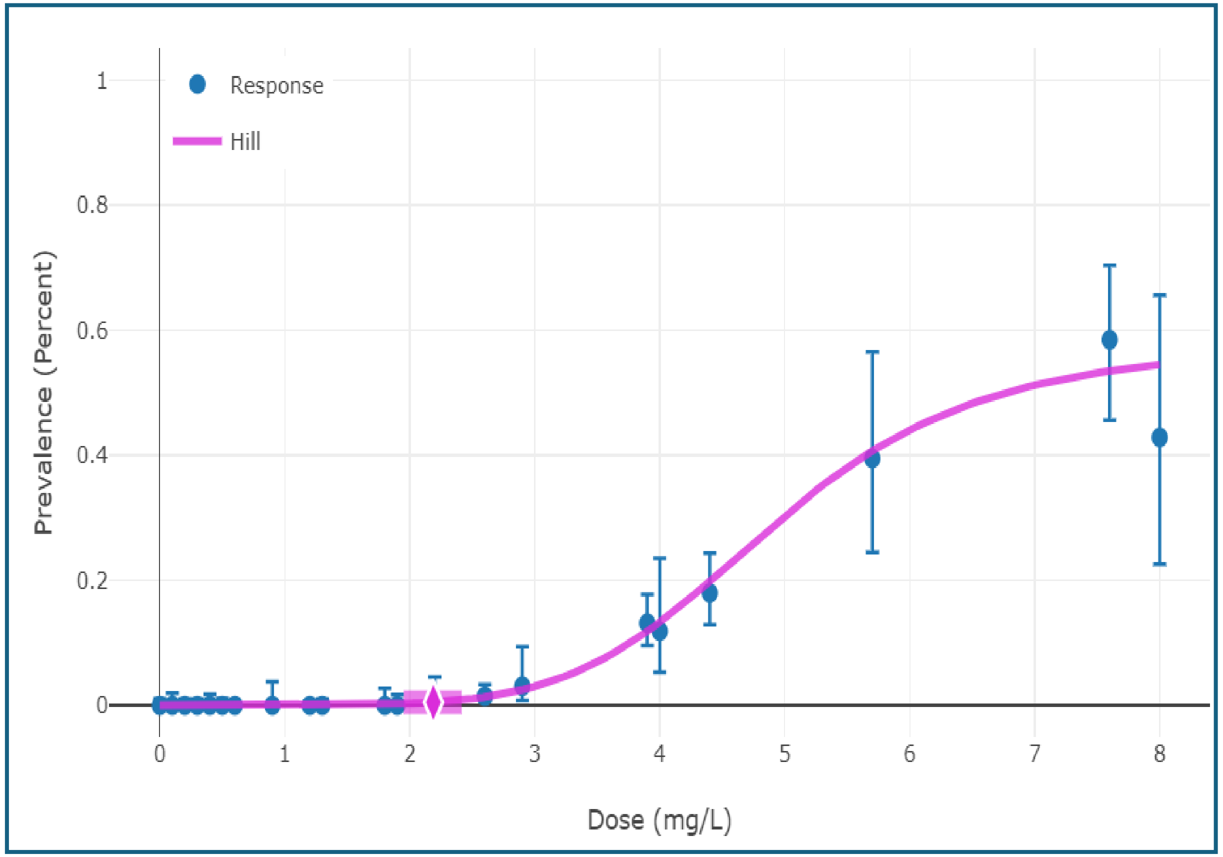

EPA analysis replicated

IQ or Cognition Score (BMR=0.5 standard deviation)

The models do not fit the data

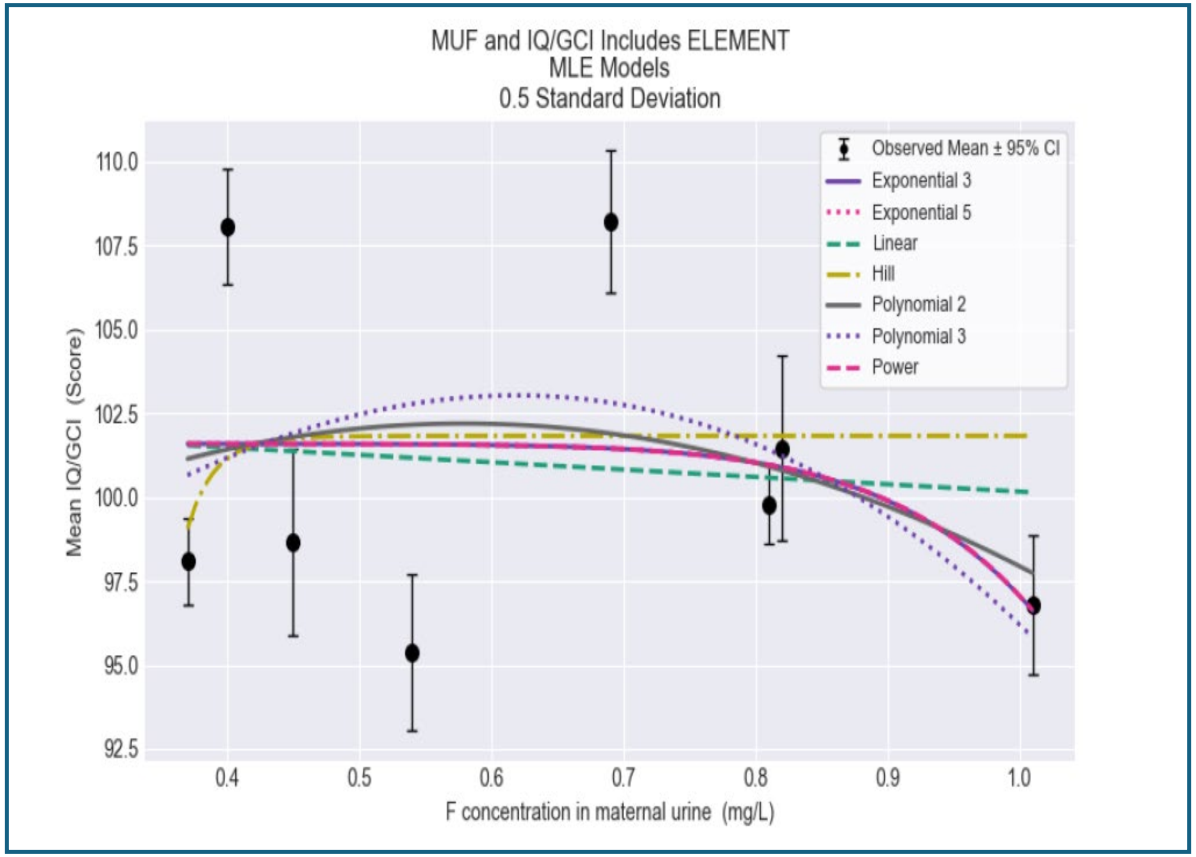

Author's Analysis
